# Grim-19 plays a key role in mitochondrial steroidogenic acute regulatory protein stability and ligand-binding properties in Leydig cells

**DOI:** 10.1016/j.jbc.2022.102671

**Published:** 2022-11-02

**Authors:** Hu Qu, Ke He, Zi-hao Zou, Gang Niu, Li Lu, Bing Yao, Wen-wen Zhong, De-juan Wang, Wei Li

**Affiliations:** 1 Department of Urology, The Sixth Affiliated Hospital, Sun Yat-Sen University, Guangzhou, Guangdong, P.R. China; 2 Department of Obstetrics and Gynecology, The First Affiliated Hospital, Sun Yat-Sen University, Guangzhou, Guangdong, P.R. China; 3 Department of Urology, The Third Affiliated Hospital of Guangzhou Medical University, Guangzhou, Guangdong, P.R. China; 4 Department of Coloproctology, The Sixth Affiliated Hospital, Sun Yat-sen University, Guangzhou, GuangDong, P.R. China; 5 Guangdong Provincial Key Laboratory of Colorectal and Pelvic Floor Diseases, The Sixth Affiliated Hospital, Sun Yat-sen University, Guangzhou, Guangdong, P.R. China; 6 Department of Human Anatomy, Histology, and Embryology, Air Force Medical University, Xi’an, P.R. China

**Keywords:** *GRIM-19*, Leydig cells, mitochondrion, StAR, steroidogenesis, phosphorylation, ECM, extracellular matrix, EDS, ethane dimethane sulfonate, GC, germ cell, IF, immunofluorescence, LC, Leydig cell, NAC, N-acetyl-L-cysteine, ROS, reactive oxygen species, TP, testosterone propionate

## Abstract

Grim-19 (gene associated with retinoid-IFN–induced mortality 19), the essential component of complex I of mitochondrial respiratory chain, functions as a noncanonical tumor suppressor by controlling apoptosis and energy metabolism. However, additional biological actions of Grim-19 have been recently suggested in male reproduction. We investigated here the expression and functional role of Grim-19 in murine testis. Testicular Grim-19 expression was detected from mouse puberty and increased progressively thereafter, and GRIM-19 protein was observed to be expressed exclusively in interstitial Leydig cells (LCs), with a prominent mitochondrial localization. *In vivo* lentiviral vector–mediated knockdown of Grim-19 resulted in a significant decrease in testosterone production and triggered aberrant oxidative stress in testis, thus impairing male fertility by inducing germ cell apoptosis and oligozoospermia. The control of testicular steroidogenesis by GRIM-19 was validated using the *in vivo* knockdown model with isolated primary LCs and *in vitro* experiments with MA-10 mouse Leydig tumor cells. Mechanistically, we suggest that the negative regulation exerted by GRIM-19 deficiency-induced oxidative stress on steroidogenesis may be the result of two phenomena: a direct effect through inhibition of phosphorylation of steroidogenic acute regulatory protein (StAR) and subsequent impediment to StAR localization in mitochondria and an indirect pathway that is to facilitate the inhibiting role exerted by the extracellular matrix on the steroidogenic capacity of LCs *via* promotion of integrin activation. Altogether, our observations suggest that Grim-19 plays a potent role in testicular steroidogenesis and that its alterations may contribute to testosterone deficiency-related disorders linked to metabolic stress and male infertility.

*GRIM-19* (gene associated with retinoid-IFN–induced mortality 19), coding for an approximately 16-kDa protein that induces apoptosis in a number of cancer cell lines, has been identified as a cell death activator involved in IFN-beta– and retinoic acid–induced cell death (1). Subsequent study has characterized GRIM-19 as an intrinsical subunit of mitochondrial NADH: ubiquinone oxidoreductase (complex I) in bovine heart, thus pointing out a novel link between the mitochondrion function and cell apoptosis (2). Homologous deletion of GRIM-19 causes embryonic lethality at embryonic day 9.5 (3), and elimination of GRIM-19 destroys the assembly and electron transfer activity of complex I and also influences the other complexes in the mitochondrial respiratory chain (4). Disruption of mitochondrial transmembrane potential (△ψm) by GRIM-19 mutants enhances the cells sensitivity to apoptotic stimuli (5). Therefore, GRIM-19 is an indispensable component of mitochondrial complex I and is essential for early embryonic development. Moreover, GRIM-19 has also been reported to be able to suppress constitutive STAT3-induced cellular transformation by downregulating the expression of a number of cellular genes involved in cell proliferation and apoptosis (6).

Notably, the biological effects of GRIM-19 known to date are mostly focused on its antiapoptotic potential and pathological relevance, that is, tumorigenesis. However, additional as yet poorly characterized physiological actions of GRIM-19 cannot be ruled out. For example, it has been demonstrated that GRIM-19 regulates Ca^2+^ homeostasis and the Ca^2+^-dependent NFAT signaling pathway, and its expression is necessary for early heart development in *Xenopus*. GRIM-19^−/−^ blastocysts display abnormal mitochondrial structure, morphology, and cellular distribution as expected (7). Furthermore, somatic missense mutations in GRIM-19 were detected in 3 of 20 sporadic Hürthle cell carcinomas of the thyroid. The latter report not only provided novel clues to the consistent linkage of increased mitochondrial number and increased cell growth that characterizes Hürthle cell tumors but also implicated a potential involvement of GRIM-19 in normal steroidogenesis (8). Nevertheless, the functional roles, if any, of GRIM-19 in such peripheral systems remain unexplored.

Testis is a complex endocrine organ in which different cell types interplay in the fine tuning of the reproductive function under the control of a plethora of endocrine, paracrine, and autocrine regulatory signals (9). Among different factors with key roles involved in testicular homeostasis, GnRH (gonadotropin-releasing hormone)–LH (luteinizing hormone)–testosterone axis plays an essential role in the regulation of testicular function (10). Emerging data of recent years have confirmed that mitochondria act as a key control point for the regulation of steroid hormone biosynthesis since the first and rate-limiting step in steroidogenesis is the transfer of cholesterol across the intermembrane space from the outer mitochondrial membrane to the inner mitochondrial membrane, a process dependent on the action of steroidogenic acute regulatory protein (StAR) (11). △ψm, mitochondrial ATP synthesis, and mitochondrial pH are all required for acute steroid biosynthesis, suggesting that mitochondria must be energized, polarized, and actively respiring to support Leydig cell (LC) steroidogenesis (12). To this end, alterations in the state of mitochondria may be involved in the direct regulation of steroid biosynthesis (13). Indeed, it has been shown recently that Grim-19 KOs display defects in testosterone biosynthesis through unidentified mechanisms (14). The identification of GRIM-19 as an essential component of complex I for maintenance of normal mitochondrial membrane potential prompted us to evaluate whether this signal is expressed in murine testis. Our current data are suggestive of a possible involvement of GRIM-19 signaling in the direct control of gonadal function in male mice, underscoring an unexpected reproductive facet of this conventional tumor suppressor.

## Results

### Characterization of Grim-19 expression in mammalian testis

**Figure 1:**
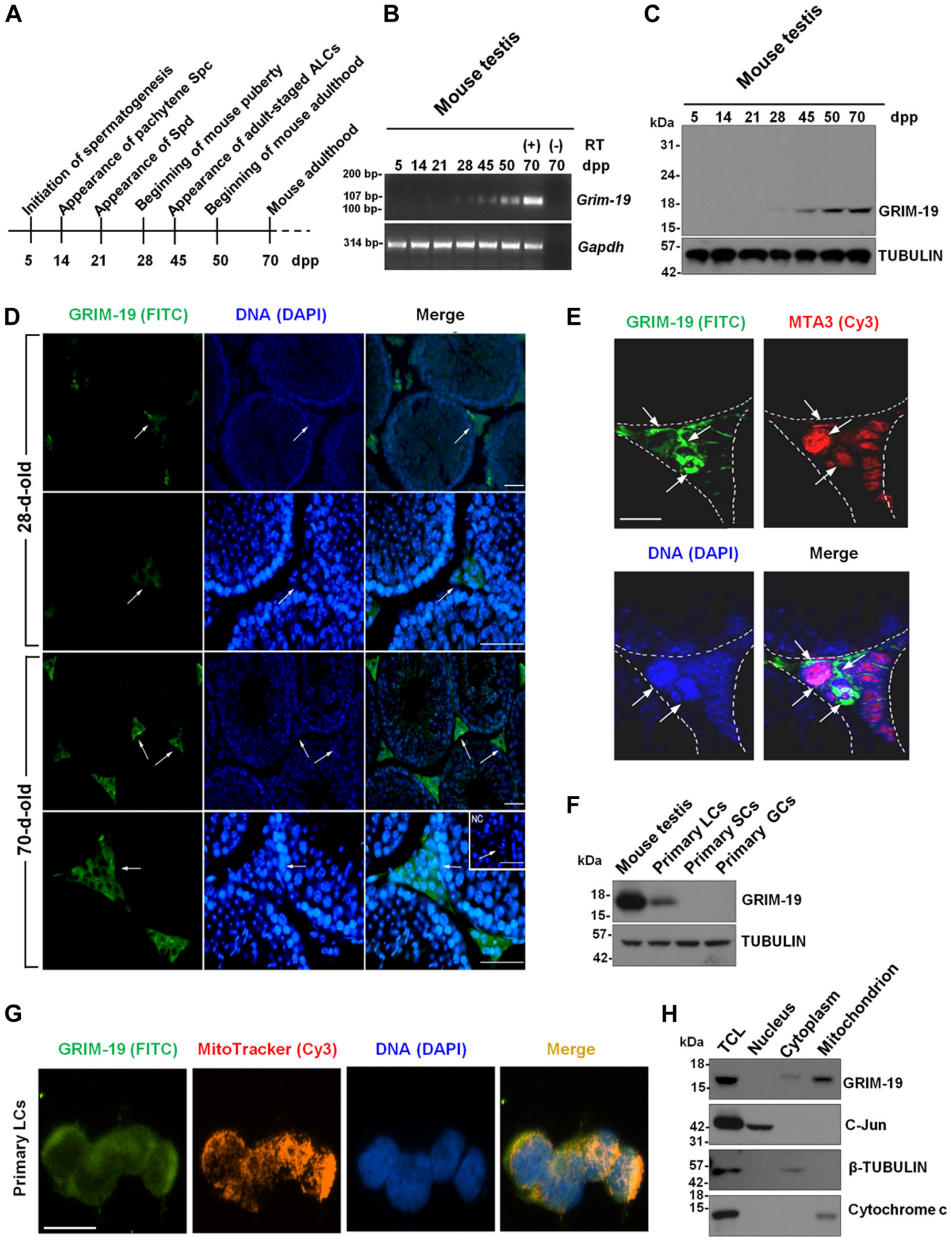
**Expression pattern of Grim-19 in rodent and human testes.***A*, diagram of the stages of mouse spermatogenesis during postnatal development. *B*, representative RT-PCR assay of the expression levels of *Grim-19* mRNA in developing mouse testes was presented. Amplification of *Gapdh* mRNA served as the internal control. PCR assay without reverse transcription (negative control) was also accompanied. *C*, immunoblotting analysis of GRIM-19 expression along the postnatal testicular development. *D*, sections of developing testis immunostained with a rabbit anti-GRIM-19 polyclonal antibody and counterstained with DAPI. Clear immunofluorescent staining is observed in the testicular interstitium (*white arrows*). Preincubation with excess immunogenic GRIM-19 peptide completely eliminated the positive staining in testis, further confirming the specificity of the primary antibody (inserted panel). NC, negative control. Bar = 25 μm *E*, representative immunostaining of GRIM-19 and MTA3 in testicular tissues from adult mice. Bar = 25 μm *F*, primary Leydig cells (LCs), Sertoli cells (SCs), and germ cells (GCs) were isolated and purified as described in Experimental procedures. Expression pattern of GRIM-19 was then verified in different spermatogenic cells and in adult mouse testis using immunoblotting. TUBULIN served as a loading control. *G*, primary LCs were labeled with MitoTracker Red, followed by immunofluorescence staining with anti-GRIM-19. Nuclei were counterstained using DAPI. Bar = 10 μm *H*, Mitochondrial, nuclear, and cytoplasmic fractions were prepared from primary LCs as described in Experimental procedures. The fractionated extracts were then subjected to immunoblotting with anti-GRIM-19 or indicated antibodies for fractionation markers. β-TUBULIN was used as a cytoplasmic marker, while C-Jun and Cytochrome c as nuclear and mitochondrial markers, respectively. DAPI, 4′,6-diamidino-2-phenylindole; TCL, total cell lysates.

Testicular expression profile of Grim-19 at different stages of postnatal development was first explored in mice. Testes were obtained from 5-, 14-, 28-, 45-, 50-, and 70-day-old mice (n = 7/group, Fig. 1*A*), corresponding to the initiation of spermatogenesis (5 days), appearance of pachytene spermatocytes (14 days), appearance of spermatids (21 days), beginning of puberty (28 days), appearance of adult-staged adult LCs (45 days), beginning of adulthood (50 days), and adult (70 days) stages of postnatal maturation, respectively (15). Persistent expression of *Grim-19* mRNA was detected from puberty (28 days) and increased progressively thereafter, with maximum values observed in adulthood (70-day-old) samples (Fig. 1*B*). Immunoblotting analysis verified such an expression profile at protein level (Fig. 1*C* and Table S1). Subsequently, the pattern of testicular expression of GRIM-19 peptide in developing testis was evaluated by immunofluorescence (IF), which demonstrated an exclusive distribution of GRIM-19 immunoreactivity within testicular interstitium (Fig. 1*D*). Specificity of GRIM-19 immunostaining was confirmed by preabsorption of the primary antibody or using nonspecific rabbit IgG, a procedure that completely blocked labeling of testis sections (inserted panel in Figs. 1*D* and S1). Additionally, GRIM-19 protein localized to LCs was visualized by MTA3 (a specific LC marker)-positive expression in adult testis using double-labeling IF (Fig. 1*E*) (15). The predominant expression of GRIM-19 in rodent LCs was further confirmed by immunoblotting in purified testicular cells (Fig. 1*F*), as well as by analysis of GRIM-19 expression using rat testis sections following selective elimination of mature LCs by systemic administration of the cytotoxic drug ethane dimethane sulfonate (EDS) (Fig. S2) (15). The subcellular localization of a protein is often tied to its function (5), so it is important to determine where our protein of interest resides. IF revealed a punctate distribution of GRIM-19 in the cytoplasm of primary LCs, which interestingly, was overlapped with Mitotracker labeling in these cells (Fig. 1*G*). We also detected a significant amount of GRIM-19 protein in the mitochondrial fraction, along with the well-known mitochondrial protein Cytochrome c (16), whereas GRIM-19 protein was weakly expressed in the cytoplasmic fraction and undetectable in the nuclear fraction of LCs (Fig. 1*H*). These findings, in accordance with previous reports which demonstrate that GRIM-19 contains several mitochondrial localization signals and acts as an essential component of mitochondrial complex I (2, 5), suggest that the biological roles of GRIM-19 are likely to be based on its function in mitochondria.

### Indispensible involvement of Grim-19 in testicular steroidogenesis and spermatogenesis

**Figure 2:**
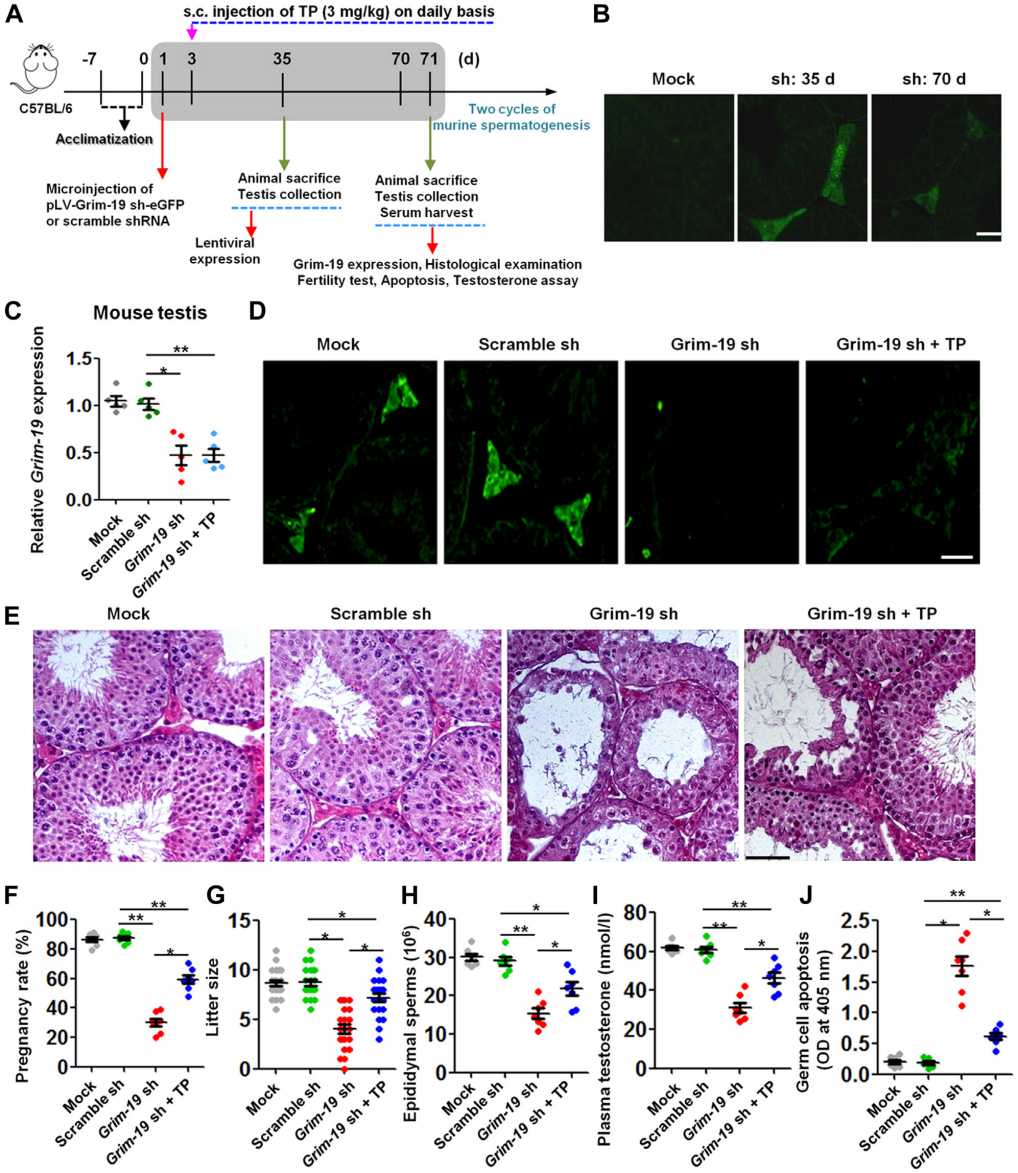
**Effects of *in vivo* knockdown of Grim-19 on testicular morphology, male fertility, epididymal sperm parameters, and germ cell apoptosis.***A*, schematic representation of the experimental procedures used in the *in vivo* lentiviral vector–mediated shRNA treatment. TP, testosterone propionate. *B*, enrichment of lentiviral shRNA in testicular interstitium following microinjection was demonstrated by observing frozen sections under a fluorescence microscope. Bar = 25 μm *C*, *Grim-19* inhibition in testis by *in vivo* shRNA treatment was confirmed using RT-qPCR at 71 days after microinjection. Data were presented as the mean ± SEM (n = 5, ∗*p* < 0.05 and ∗∗*p* < 0.01). *D*, GRIM-19 immunostaining in testicular sections was carried out 71 days after microinjection. Bar = 25 μm. TP, testosterone propionate. sh, lentiviral shRNA. *E*, representative H&E–stained transverse testis sections showing morphological changes at 71 days after microinjection. Bar = 25 μm *F* and *G*, fertility test: one male mouse was mated with two WT female mice. Upon detection of copulatory plugs the following morning, the females with copulatory plugs were replaced with another WT female mice. The mating period consisted of 18 days. The pregnancy rate (pregnancy/vaginal plug formation) is expressed as a percentage (n = 7, ∗*p* < 0.05 and ∗∗*p* < 0.01). Little size was recorded accordingly (n = 9 for male mice, ∗*p* < 0.05). *H*, quantification of the concentrations of caudal spermatozoa at 71 days after microinjection (n = 7, ∗*p* < 0.05 and ∗∗*p* < 0.01). *I*, plasma testosterone levels (nmol/l) in mice from different groups at 71 days after microinjection were assessed using an ELISA method (n = 10, ∗*p* < 0.05 and ∗∗*p* < 0.01). *J*, the apoptotic status in Ctrl- or Grim-19 shRNA-treated testes was evaluated in triplicate spectrophotometry using apoptotic ELISA at 405 nm (n = 7, ∗*p* < 0.05 and ∗∗*p* < 0.01). RT-qPCR, reverse transcription quantitative PCR.

To elucidate the biological roles of Grim-19, we employed a previously validated lentiviral microinjection approach to inhibit Grim-19 expression *in vivo*. About 20 μl of pLV-Grim-19 sh-eGFP or scramble shRNA (7 ng of viral capsid proteins/μl) were microinjected into testicular interstitium using a fiber optics probe under a dissecting microscope. Mice were then allowed to recover and were sacrificed at day 71 after shRNA injection (Fig. 2*A*). This time point was chosen because one cycle of murine spermatogenesis consists of 35 days (17). Using a GFP reporter allele to evaluate the targeting efficiency and specificity (18), we found that lentivirus-mediated bioluminescence was specifically enriched in the testicular interstitium at day 35 and day 70 following microinjection, compared to a negative expression of bioluminescence in mock controls (Fig. 2*B*). Relative to Scramble sh-treated testes, treatment with Grim-19 shRNA resulted in a ∼53.8% reduction in the expression levels of *Grim-19* mRNA (*p* < 0.05), and Grim-19 sh-mediated inhibition of Grim-19 expression could not be rescued by supplement with exogenous testosterone (testosterone propionate [TP], Fig. 2*C*). The effectiveness of inhibition of Grim-19 by shRNA, as well as the inability of exogenous testosterone to rescue the expression of GRIM-19 in shRNA-treated testis, was further confirmed at protein level by immunostaining (Fig. 2*D*). The apparent inhibition of Grim-19 in LCs made us curious whether Grim-19 deficiency has a role in affecting male fertility, which was then investigated by assessment of a qualitative endpoint (*i.e*., morphological changes), as well as several quantitative endpoints (*i.e*., pregnancy, litter size, density and mobility of epididymal sperm, levels of plasma testosterone, and testicular apoptosis). Histological examination revealed morphological defects in Grim-19 shRNA-treated testes, including thinning of seminiferous epithelium, desquamation of germ cells (GCs), and scarcity of mature sperm (Fig. 2*E*). Consequently, the mice treated with Grim-19 shRNA showed remarkable defects in male fertility, relative to their respective Scramble sh-treated counterparts (29.9 ± 6.8 *versus* 87.6 ± 3.4 pregnancy rate for Grim-19 sh *versus* Scramble sh, *p* < 0.01, Figure 2*F*; 4.0 ± 2.2 *versus* 8.8 ± 1.5 litter size for Grim-19 sh *versus* Scramble sh, *p* < 0.05, Figure 2*G*; 15.3 ± 3.6 *versus* 29.1 ± 2.7 epididymal sperm × 10^6^ for Grim-19 sh *versus* Scramble sh, *p* < 0.01, Fig. 2*H*). The defects in male fertility were probably caused by testosterone deficiency-induced GC apoptosis, evidenced by hormonal and biochemical analysis (31.0 ± 6.5 *versus* 60.9 ± 4.1 plasma testosterone for Grim-19 sh *versus* Scramble sh, *p* < 0.01, Figure 2*I*; 1.8 ± 0.4 *versus* 0.2 ± 0.1 germ cell apoptosis for Grim-19 sh *versus* Scramble sh, *p* < 0.01, Fig. 2*J*). The latter hypothesis becomes more feasible when supplement with TP effectively ameliorated reproductive defects in Grim-19 shRNA-treated mice (Fig. 2, *E*–*J*). Thus, Grim-19 inhibition in LCs yields a progressive testis degeneration resulting from a disrupted testicular steroidogenesis.

### Validation of the role of Grim-19 in testicular steroidogenesis using *in vitro* experiments with primary LCs and MA-10 cells

**Figure 3:**
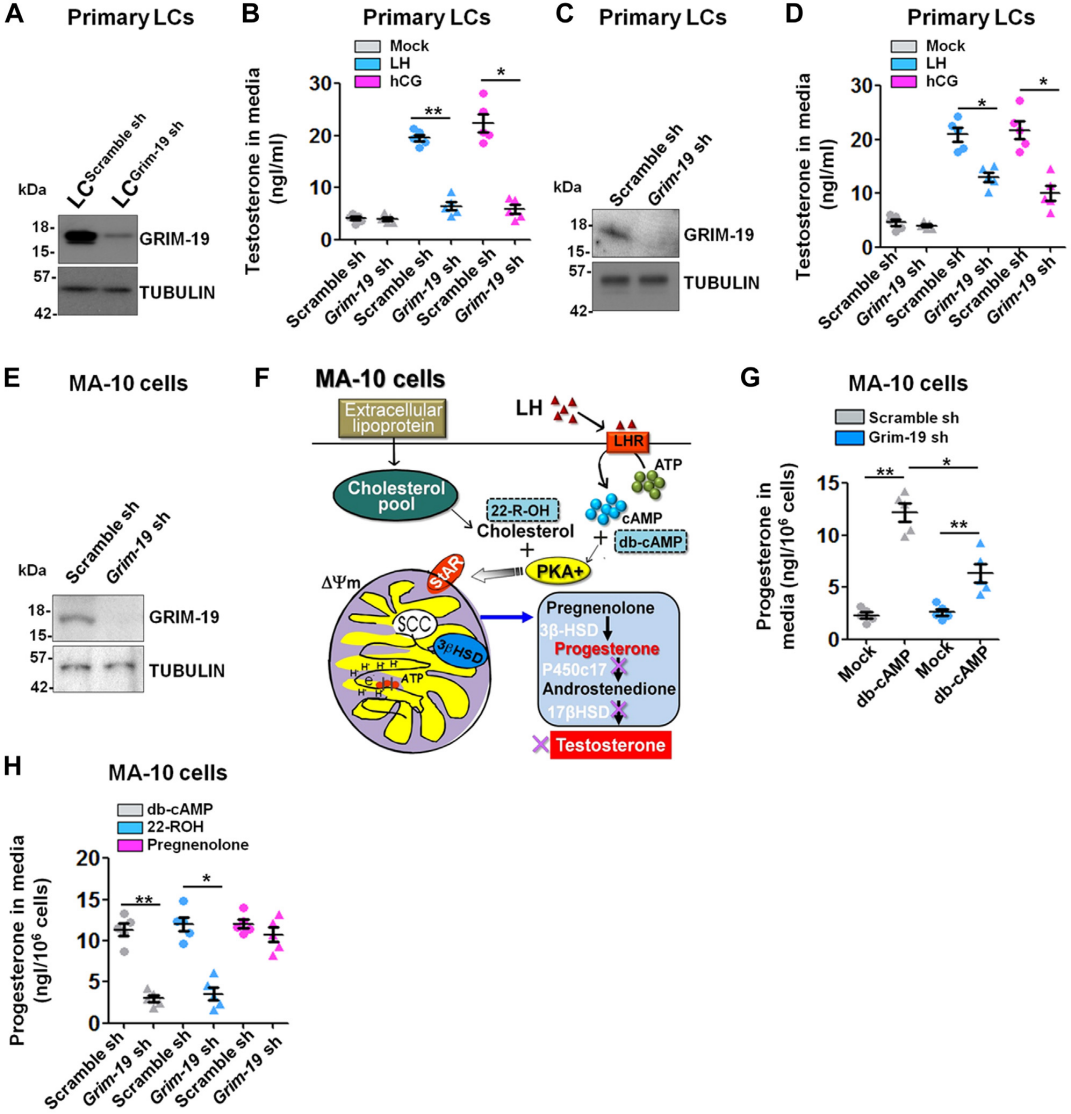
**Effects of Grim-19 knockdown on basal and gonadotropin-stimulated steroidogenesis in primary LCs and MA-10 cells.***A*, primary LCs were isolated and purified from Scramble sh or *Grim-19* sh-treated testes at 71 days after microinjection. The knockdown efficiency in testis was confirmed using immunoblotting. *B*, primary LCs^Grim-19 sh^ or Ctrl LCs were treated with 100 ng/ml luteinizing hormone (LH)/human chorionic gonadotropin (hCG) for 12 h, followed by measurement of testosterone concentration in the culture media using an ELISA method (n = 5, ∗*p* < 0.05 and ∗∗*p* < 0.01). *C*, transient knockdown of Grim-19 expression in primary LCs from WT mice was verified using immunoblotting at 48 h after transfection. *D*, 48 h after transfection, primary LCs were treated with 100 ng/ml LH/hCG for 12 h, followed by measurement of testosterone concentration in the culture media using an ELISA method (n = 5, ∗*p* < 0.05). *E*, the establishment of the MA-10^Grim-19−/−^ cells was confirmed using immunoblotting. *F*, schematic presentation depicting the main metabolic pathways required for progesterone synthesis in MA-10 cells. *G*, MA-10^Grim-19−/−^ or control cells were treated with 1 mM dibutyryl-cAMP (db-cAMP) for 12 h, followed by measurement of progesterone concentration in the culture medium using an ELISA method (n = 5, ∗*p* < 0.05 and ∗∗*p* < 0.01). *H*, progesterone production stimulated for 12 h by db-cAMP (1 mM), 22R-hydroxycholesterol (22-ROH, 5 μM), or pregnenolone (5 μM) in the MA-10^Grim-19−/−^ or control cells was determined as described. Quantitative values are means ± S.E.M. (n = 5, ∗*p* < 0.05 and ∗∗*p* < 0.01). LC, Leydig cell.

To better understand the molecular events underlying the disruption in testicular steroidogenesis in Grim-19-deficient males, we isolated and purified primary LCs from Scramble sh and Grim-19 sh-treated testes (Figs. S3 and3*A*). Primary LCs were then challenged for 12 h with 100 ng/ml LH/hCG. Relative to LC^Scramble sh^, the stimulated testosterone concentration in culture media was significantly reduced in LC^Grim-19 sh^ (6.4 ± 1.8 *versus* 19.5 ± 1.4 LH treatment for Grim-19 sh *versus* Scramble sh, *p* < 0.01; 5.8 ± 1.9 *versus* 22.4 ±3.9 hCG treatment for Grim-19 sh *versus* Scramble sh, *p* < 0.05), whereas ablation of Grim-19 had no effects on steroidogenesis at the resting basal state in both cells (Fig. 3*B*). To determine whether transient Grim-19 knockdown could also affect testosterone synthesis, we transfected primary LCs from WT testes with Scramble sh and Grim-19 sh (Fig. 3*C*). In line with the findings obtained from *in vivo* knockdown model with isolated primary LCs, knockdown of Grim-19 also attenuated the gonadotropin-induced testosterone production in LCs but had no effects on steroidogenesis at the resting basal state (Fig. 3*D*). To further define functional details of Grim-19, we generated the MA-10^Grim-19−/−^ cells (Fig. 3*E*). As a relatively homogeneous clonal strain of mouse Leydig tumor cells, MA-10 cells have been proved to be a suitable model system for the study of the regulation of differentiated functions of LCs and gonadotropin actions. MA-10 cells retain many of the properties of LCs except that the production of androgens by these cells is almost undetectable due to lack of 17α-hydroxylase/17,20 lyase/17,20 desmolase expression and activity. Thus, the study on the steroidogenesis by using MA-10 is more precise to be referred as progesterone but not testosterone production (Fig. 3*F*) (15). Consistent with the results obtained from primary LCs, relative to MA-10^Scramble sh^, the db-cAMP-stimulated progesterone concentration in culture media was significantly decreased in MA-10^Grim-19 sh^ (6.4 ± 2.0 *versus* 12.2 ± 1.9 for Grim-19 sh *versus* Scramble sh, *p* < 0.05, Fig. 3*G*). To identify at which step the steroidogenic process was impaired by Grim-19 deficiency, MA-10^Grim-19 sh^ cells were challenged with different stimulus including db-cAMP, 22-ROH, or pregnenolone. Intriguingly, a noticeable inhibition of progesterone synthesis was still observed when db-cAMP or 22-ROH was used as a substrate. In contrast, the inhibitory effect of Grim-19 deficiency was totally reversed by the addition of pregnenolone in MA-10^Grim-19 sh^ cells (Fig. 3*H*). Of note, Grim-19 deficiency had no effects on cell viability and apoptosis, as revealed by methyl thiazolyl tetrazolium and apoptotic ELISA assays (Fig. S4). Thus, at least one inhibitory effect of Grim-19 deficiency on the stimulated steroidogenesis appears to occur at the steroidogenic acute regulatory protein (StAR) step.

### Indirect control of phosphorylation of StAR by GRIM-19 in stimulated LCs

**Figure 4:**
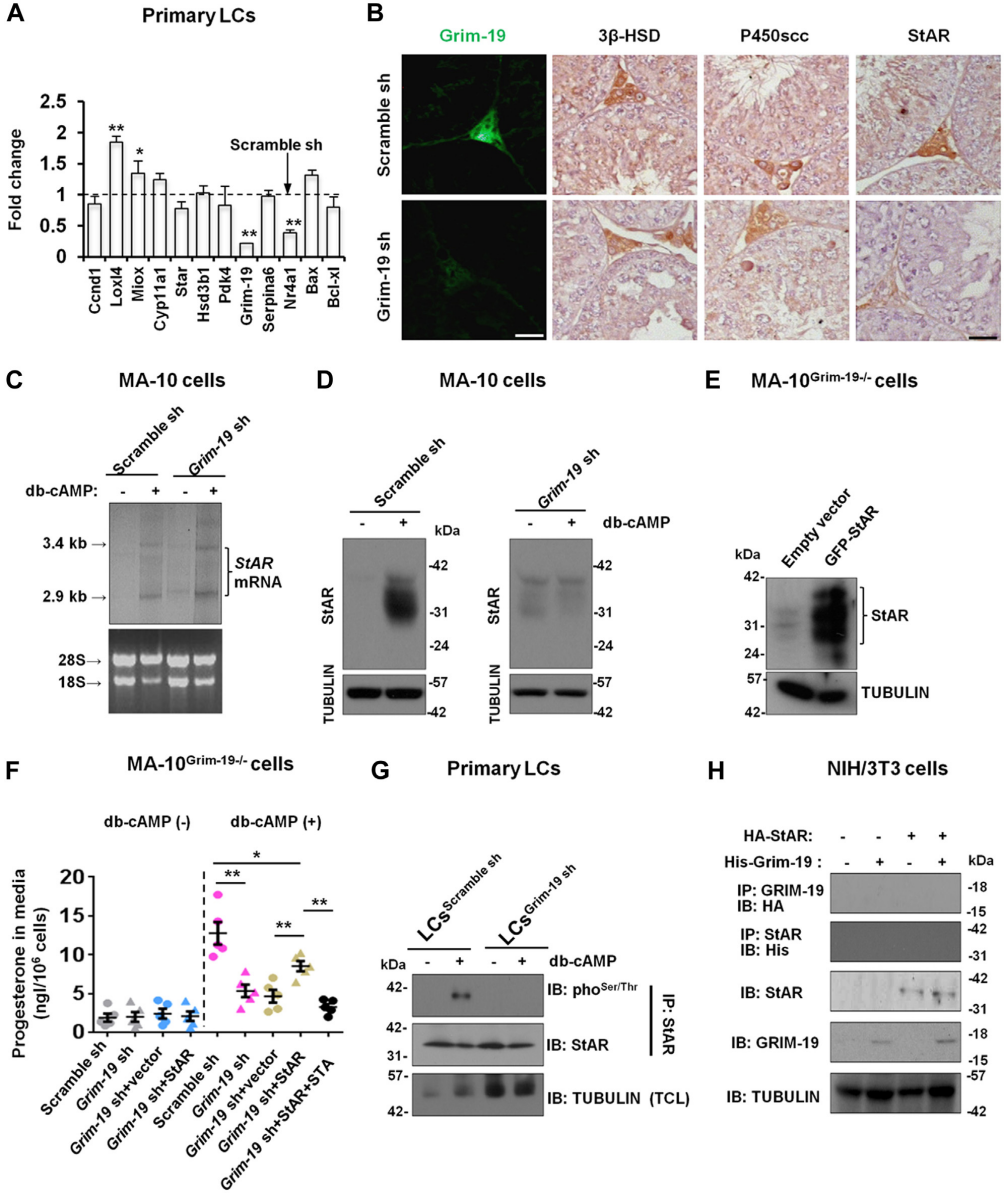
**Grim-19 depletion impairs the stability of StAR by modulating its phosphorylation in db-cAMP-stimulated LCs.***A*, expression levels of different key factors essential for testicular functions in primary LCs isolated from Grim-19 shRNA-treated testis were determined using qPCR. ∗*p* < 0.05 and ∗∗*p* < 0.01 compared to the values in primary LCs isolated from Scramble shRNA-treated testis (*dash line*). *B*, expression levels of different key factors essential for testicular steroidogenesis, as well as expression of GRIM-19, in *Grim-19* shRNA-treated testis were determined by immunohistochemical and immunofluorescent staining. Bar = 25 μm *C*, MA-10 cells with different transfections were treated with 1 mM db-cAMP for 12 h, followed by nonradioactive Northern blot analysis to measure the changes in *StAR* mRNA expression. The *18S* and *28S* stained with ethidium bromide demonstrated the presence of comparable amounts of RNA samples in all lanes. *D*, MA-10^Grim-19−/−^ or control cells were treated with 1 mM dibutyryl-cAMP (db-cAMP) for 12 h, followed by immunoblotting analysis. *E*, MA-10^Grim-19−/−^ cells were transfected with a mouse StAR ORF expression plasmid with Lipofectamine 3000 for 48 h. The overexpression of StAR was then confirmed using immunoblotting analysis. *F*, MA-10 cells with different transfections were treated with 1 mM db-cAMP for 12 h, followed by measurement of progesterone concentration in the culture medium using an ELISA method (n = 5, ∗*p* < 0.05 and ∗∗*p* < 0.01). *G*, primary LCs were isolated and purified from Scramble sh or *Grim-19* sh-treated testes at 71 days after microinjection. Cells were then treated with 1 mM db-cAMP for 12 h, followed by sequential immunoprecipitation (IP) and immunoblotting analysis using antibodies as indicated. *H*, NIH/3T3 cells were transfected with His-Grim19 or HA-StAR. At 48 h posttransfection, cells were lysed and subjected to IP and immunoblotting analysis using the indicated antibodies. LC, Leydig cells; qPCR, quantitative PCR.

As further exploration of the core signaling pathways responsible for Grim-19 action in stimulated steroidogenesis, we examined expression levels of several genes known to be essential for LC biology (*Ccnd1*, cell cycle progress and proliferation; *Hsd3b1*, testosterone biosynthesis; *Loxl4*, metal ion binding and oxidoreductase activity; *Cyp11a1*, steroid synthesis; *Pdk4*, ATP binding and pyruvate dehydrogenase kinase activity; *Serpina6*, lipid and steroid binding; *Star*, transport of cholesterol from the outer mitochondrial membrane to the inner mitochondrial membrane; *Miox*, NADP binding, metal ion binding, and oxidoreductase activity; *Nr4a1*, transcription regulator activity; *Bax*, proapoptotic gene; *Bcl-xl*, antiapoptotic gene) (19, 20), in primary LCs isolated from Scramble sh or Grim-19 sh-treated testes. Relative to LCs from Scramble sh-treated testes, *Loxl4* and *Miox* transcripts were significantly increased while *Nr4a1* transcripts were noticeably decreased in LCs isolated from Grim-19 sh-treated testes (Fig. 4*A*). We speculated, therefore, that ablation of Grim-19 may impair steroidogenesis (*Nr4a1* is an androgen-regulated gene (21)) and augment oxidative stress but has no effects on cell proliferation, apoptosis, or cellular metabolism of metal ion. We then evaluated the expression levels of steroidogenic enzymes known to be essential for testicular steroidogenesis by means of immunostaining. StAR was the only enzyme that was observed to be significantly downregulated in Grim-19 sh-treated testes compared to Scramble sh-treated testes (Fig. 4*B*). Therefore, it is very likely that Grim-19 deficiency may affect posttranslational modification of StAR protein in LCs. Upon intracellular pulses of cAMP, StAR is rapidly synthesized and then regulates fundamentally the transfer of cholesterol from outside into mitochondria, thus serving as a key factor mediating the acute steroidogenesis (22). Intriguingly, db-cAMP treatment of Scramble sh or Grim-19 sh-treated MA-10 cells induced the expression of the 3.4-kb precursor form of *StAR* mRNA as well as the 2.9-kb processed form in both cells (Fig. 4*C*). Contrarily, db-cAMP induced the expression of the 37-kDa precursor form of StAR protein as well as the 30-kDa processed form in Scramble sh-treated MA-10 cells but failed to do so in Grim-19 sh-treated MA-10 cells (Fig. 4*D*). To further confirm that the inhibitory effect of Grim-19 deficiency on steroidogenesis occurs at the level of StAR from a functional standpoint, we transiently overexpressed exogenous StAR in the MA-10^Grim-19−/−^ cells (Fig. 4*E*). Relative to empty vector–transfected MA-10^Grim-19−/−^ cells, overexpression of StAR significantly rescued db-cAMP stimulated progesterone production in the MA-10^Grim-19−/−^ cells, while ablation of Grim-19 or overexpression of StAR had no effects on basal progesterone production in MA-10 cells. By contrast, pretreatment with the broad serine/threonine kinase inhibitor staurosporine (STA) substantially blocked the StAR overexpression-rescued progesterone production in MA-10^Grim-19−/−^ cells (Fig. 4*F*). Because phosphorylation of StAR protein plays an essential role in the modulation of localization and steroidogenic activity of StAR (23, 24), we sought to determine whether Grim-19 deficiency affects phosphorylation of StAR in stimulated LCs. Treatment of LCs^Scramble sh^ with db-cAMP significantly enhanced phosphorylation of StAR in WT LCs, and this stimulatory effect was totally abolished in LCs^Grim-19 sh^ (Fig. 4*G*). To test whether Grim-19 is directly involved in phosphorylation of StAR, we examined the interaction between StAR and GRIM-19. The reciprocal coimmunoprecipitation experiments in NIH/3T3 cells overexpressing both StAR and GRIM-19 demonstrated that there was no direct interaction between StAR and GRIM-19 (Fig. 4*H*). Together, GRIM-19 may regulate the phosphorylation of StAR in an indirect manner during gonadotropin-stimulated steroidogenesis.

### Indirect control of phosphorylation of StAR by GRIM-19 in stimulated LCs

**Figure 5:**
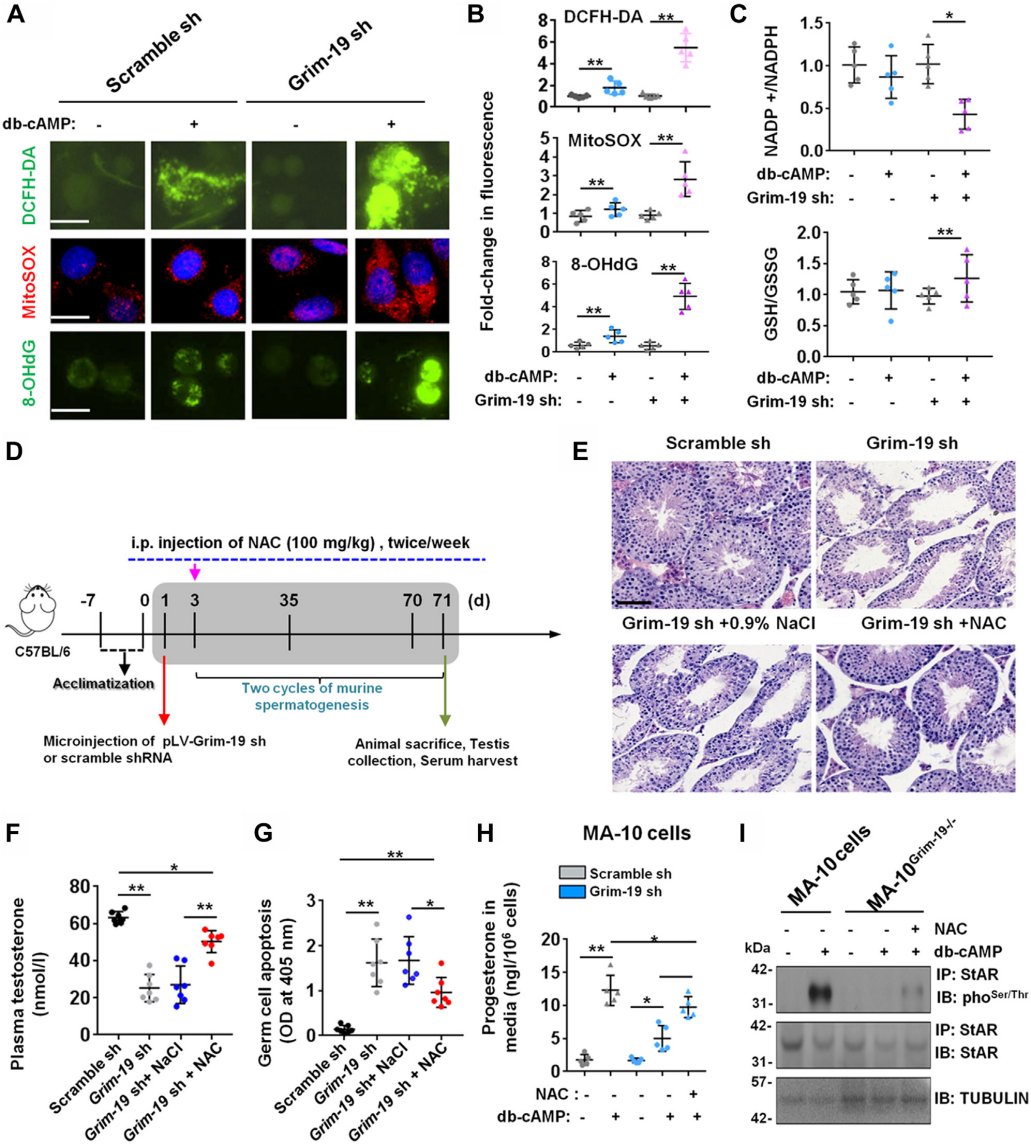
**ROS scavenger reverses GRIM-19 deficiency-impaired testicular steroidogenesis *in vitro* and *in vivo*.***A*, GRIM-19 deficiency causes aberrant oxidative stress in db-cAMP-stimulated LCs. Intracellular ROS (DCFH-DA) and mROS generations, as well as a DNA damage marker 8-oxodeoxyguanosine (8-OHdG), were detected by IF staining. Bar = 10 μm *B*, the fluorescent spectrometer analysis was carried out using 495 nm excitation and 527 nm emission filters (n = 5, ∗*p* < 0.05 and ∗∗*p* < 0.01). *C*, NADP+/NADPH and GSH/GSSG ratios were analyzed in GRIM-19 deficient LCs at 12 h after challenge with 1 mM db-cAMP (n = 5, ∗*p* < 0.05 and ∗∗*p* < 0.01). *D*, schematic representation of the Experimental procedures used in the *in vivo* lentiviral vector–mediated shRNA plus ROS scavenger treatment. *E*, representative H&E–stained transverse testis sections showing morphological changes at 71 days after microinjection and NAC cotreatment. Bar = 25 μm *F*, plasma testosterone levels (nmol/l) in mice from different groups at 71 days after microinjection were assessed using an ELISA method (n = 7, ∗*p* < 0.05 and ∗∗*p* < 0.01). *G*, the apoptotic status in testes from different experimental groups was evaluated in triplicate spectrophotometry using apoptotic ELISA at 405 nm (n = 7, ∗*p* < 0.05 and ∗∗*p* < 0.01). *H*, MA-10^Grim-19−/−^ or control cells were treated with 1 mM db-cAMP for 12 h, in the presence or absence of 5 mM of NAC, followed by measurement of progesterone concentration in the culture medium using an ELISA method (n = 5, ∗*p* < 0.05 and ∗∗*p* < 0.01). *I*, MA-10^Grim-19−/−^ or control cells were treated with 1 mM db-cAMP for 12 h, in the presence or absence of 5 mM of NAC, followed by sequential IP and immunoblotting analysis using antibodies as indicated. IF, immunofluorescence; IP, immunoprecipitation; NAC, N-acetyl-L-cysteine; ROS, reactive oxugen species.

Given that GRIM-19 is a core subunit of the mitochondrial complex I, we asked whether aberrant oxidative stress was involved in GRIM-19 deficiency-disrupted steroidogenesis. Evidenced by IF, ablation of GRIM-19 in primary LCs significantly enhanced intracellular reactive oxygen species (ROS) (DCFH-DA) and mROS (MitoSOX) productions, as well as 8-hydroxydeoxyguanosine (8-OHdG) expression, a biomarker consequent to oxidative stress (25) (Fig. 5, *A* and *B*). Consistently, GRIM-19 deficiency noticeably reduced the NADP+/NADPH ratio but induced the GSH/GSSG ratio in stimulated primary LCs (Fig. 5*C*). Thus, GRIM-19 deficiency triggers aberrant oxidative stress by ROS generation in LCs. To elucidate the essential involvement of ROS in GRIM-19 deficiency-driven defects of steroidogenesis, we administrated the ROS scavenger N-acetyl-L-cysteine (NAC) intervention in our *in vivo* knockdown model, to evaluate whether ROS scavenger could ameliorate GRIM-19 deficiency-disrupted steroidogenesis (Fig. 5*D*). Intriguingly, supplement with NAC significantly restored epithelial thickness of seminiferous tubule (Fig. 5*E*), and effectively but partially increased plasma testosterone levels (Fig. 5*F*) and reduced GC apoptosis (Fig. 5*G*), in Grim-19 sh-treated testes. Similarly in MA-10 cell cultures, coincubation with NAC markedly but partially improved progesterone synthesis in the presence of db-cAMP stimulation (Fig. 5*H*). To further clarify the critical role of ROS in GRIM-19 deficiency-driven defects of steroidogenesis, we determined whether ROS inhibition could reverse GRIM-19 deficiency-driven phosphorylation of StAR. As expected, NAC cotreatment markedly but partially restored p-StAR levels in db-cAMP-challenged MA-10^Grim-19−/−^ cells (Fig. 5*I*). Thus, GRIM-19 deficiency, may at least partially, induce aberrant phosphorylation of StAR *via* ROS-dependent manner.

### GRIM-19 deficiency potentiates adhesion of integrin-expressing MA-10 cells to distinct extracellular matrix components

**Figure 6:**
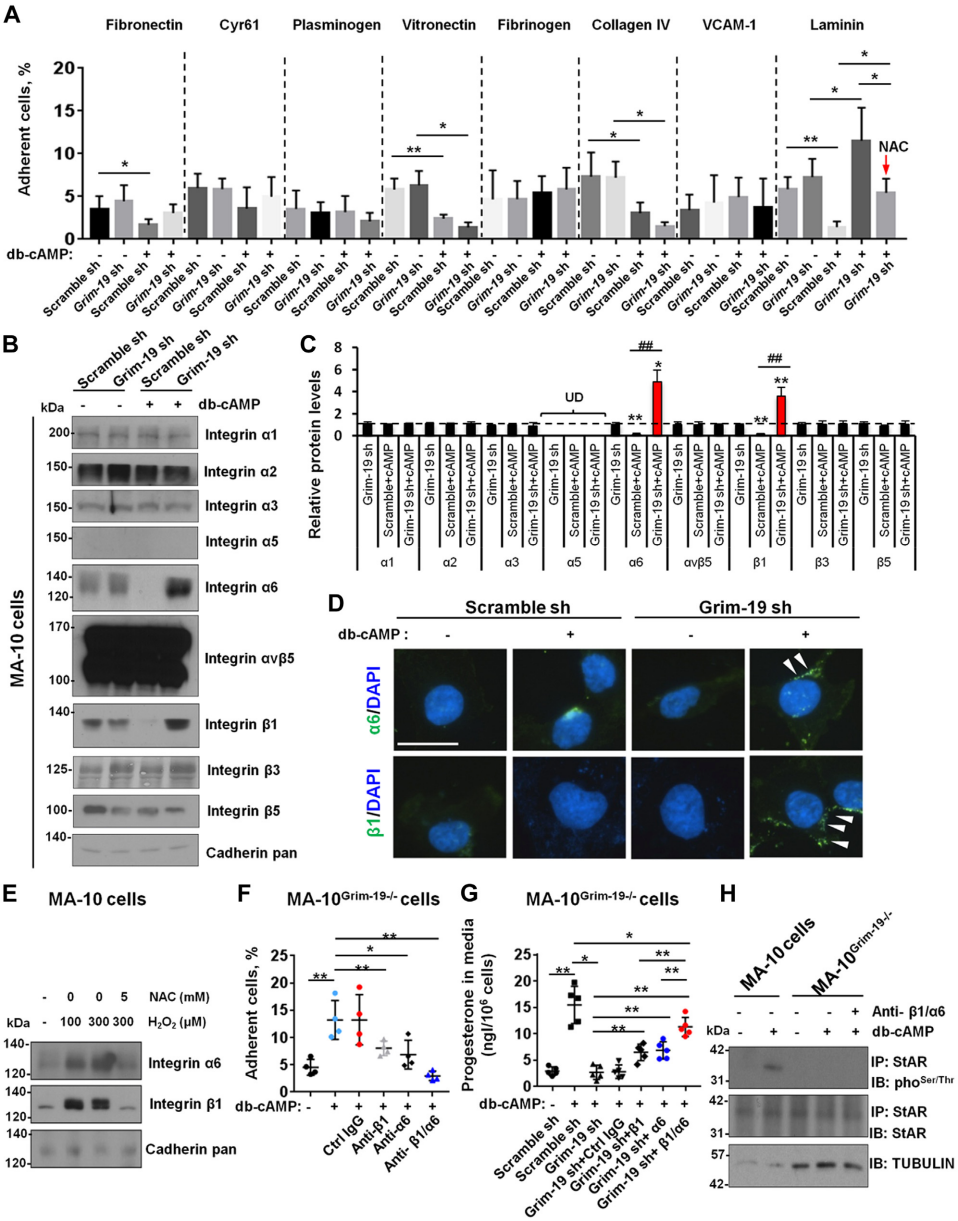
**Augmentation of ligand-binding properties in LCs by GRIM-19 deficiency during stimulated steroidogenesis.***A*, aliquots (50 μl) of 5 × 10^5^/ml MA-10 cells in DMEM/F-12 were added to the wells precoated with different ligands. Cells were challenged for 12 h with 1 mM db-cAMP, in the presence or absence of 5 mM of NAC, followed by incubation for 30 min at 37 °C. The nonadherent cells were removed by two washes with PBS and fluorescence was measured using a CytoFluor II fluorescence plate reader (n = 5, ∗*p* < 0.05 and ∗∗*p* < 0.01). *B*, MA-10^Grim-19−/−^ or control cells were treated with 1 mM db-cAMP for 12 h. Cell surface proteins were then isolated with the aid of a Cell Surface Protein Isolation Kit from Abcam, followed by immunoblotting using antibodies against individual subunits. *C*, densitometric scanning of immunoblots was performed in which the level of a target protein was normalized against the protein level in Scramble sh-treated MA-10 cells (without cotreatment with db-cAMP), which was arbitrarily set at 1 (*dash line*). Each bar represents the means ± SEM of results from three experiments using different batches of cells. Each experiment had replicate cultures (n = 4, ∗*p* < 0.05, ∗∗*p* < 0.01, and ^##^*p* < 0.01). *D*, cell surface localization of Integrin α6 and β1 subunits (*white arrow heads*) in different cells was revealed by IF staining. Bar = 10 μm *E*, MA-10 cells were treated with different doses of H_2_O_2_ as indicated, in the presence or absence of 5 mM of NAC, followed by immunoblotting. *F*, MA-10^Grim-19−/−^ cells were preincubated for 20 min at 22 °C with anti-β1 antibody (1:500 dilution), anti-α6 antibody (1:500 dilution), or with combinations of anti-β1 and anti-α6 antibodies. Cells were then added to microtiter wells coated with Laminin (5 μg/ml) that produce maximal adhesion, and cell adhesion was determined as aforementioned (n = 4, ∗*p* < 0.05 and ∗∗*p* < 0.01). *G*, MA-10^Grim-19−/−^ cells were preincubated for 20 min at 22 °C with anti-β1 antibody (1:500 dilution), anti-α6 antibody (1:500 dilution), or with combinations of anti-β1 and anti-α6 antibodies. Cells were then treated with 1 mM db-cAMP for 12 h, followed by measurement of progesterone concentration in the culture medium using an ELISA method (n = 5, ∗*p* < 0.05 and ∗∗*p* < 0.01). *H*, MA-10^Grim-19−/−^ or control cells were preincubated for 20 min at 22 °C with combinations of anti-β1 and anti-α6 antibodies as aforementioned. Cells were then treated with 1 mM db-cAMP for 12 h, followed by sequential IP and immunoblotting analysis using antibodies as indicated. IF, immunofluorescence; IP, immunoprecipitation; LC, Leydig cell.

LCs reside in the testicular interstitium where a large set of locally produced factors (*e.g.*, extracellular matrix [ECM] proteins) act in a paracrine and/or autocrine way to modulate fundamentally steroidogenesis by LCs (26). To test the possibility that GRIM-19 deficiency may alter adhesion of LCs to adjacent ECM proteins, we investigated the ability of the MA-10^Grim-19−/−^ cells to adhere to several EMC proteins. Relative to Scramble sh-treated MA-10 cells, MA-10^Grim-19−/−^ cells tended to unadhere to wells coated with fibronectin, vitronectin, or collagen IV but tended to adhere to wells coated with laminin under db-cAMP-stimulating conditions. By contrast, cotreatment with NAC substantially compromised GRIM-19 deficiency-elicited adhesion to Laminin in db-cAMP-challenged MA-10^Grim-19−/−^ cells (Fig. 6*A*). Because the subfamily of integrins comprises a monophyletic group of closely related glycoproteins critically involved in cell–ECM interactions (27) and because integrins play a key role in transduction of signals controlling testosterone production (28, 29), one can suppose that modifications in expression of these receptors underlie the found difference between the analyzed cell lines. Indeed, immunoblotting showed that Scramble sh-treated MA-10 and MA-10^Grim-19−/−^ cells strongly differ in the spectrum of expressed integrins. Upon db-cAMP challenge, laminin-specific integrin α6 and β1 were significantly downregulated in Scramble sh-treated MA-10 cells but markedly induced in MA-10^Grim-19−/−^ cells (Fig. 6, *B* and *C*). In agreement, enhancement of collagen-specific integrin α6β1 expression was observed in cell membrane by means of IF (Fig. 6*D*). The observed changes in integrin expression might be considered as a resultant feature related to ROS mechanisms because cotreatment with NAC totally abolished H_2_O_2_-induced α6β1 expression in MA-10 cells (Fig. 6*E*). To determine a relative contribution of the integrins on LCs to these adhesion and steroidogenesis processes, we examined the effect of antibodies directed against anti-α6 and anti-β1 integrin subunits using MA-10^Grim-19−/−^ cells. As shown in Figure 6*F*, MA-10^Grim-19−/−^cells efficiently migrated toward laminin-coated well, and this process depended on both α6 and β1 because anti-α6 and anti-β1 monoclonal antibodies (mAbs) both blocked the response, while combination of anti-α6 and anti-β1 mAbs completely blocked migration of MA-10^Grim-19−/−^ cells on laminin. Consequently, treatment with anti-α6 or anti-β1 mAbs both effectively improved stimulated steroidogenesis in MA-10^Grim-19−/−^ cells, with the most dramatic rescuing effects been observed in combination of two mAbs (Fig. 6*G*). By contrast, blockage with anti-α6β1 mAbs had no effects on phosphorylation of StAR protein in db-cAMP-challenged MA-10^Grim-19−/−^ cells (Fig. 6*H*). Together, inhibition of GRIM-19 induced α6β1 activation, thus controlling cell adhesion to distinct ECM component and negatively affecting steroidogenesis in stimulated LCs.

## Discussion

As an essential component of mitochondrial complex I, GRIM-19 plays a pivotal role in the regulation of mitochondrial membrane potential and dynamics (5), and mice deficient in *Grim-19* display complex I assembly disruption and embryonic lethality, emphasizing an indispensable role of Grim-19 in the maintenance of mitochondrial homeostasis (30). Previous clinical observations using spermatozoa samples from patients with asthenozoospermia, as well as a recent animal study using *Grim-19* KO mice, suggest that Grim-19 is functionally involved in the control of male reproduction, a function that appears to be mediated, at least partially, by its ability to modulate steroidogenesis and intracellular ROS (31, 32). However, the testicular cell types expressing GRIM-19 and its precise role during spermatogenesis and its possible interaction with autocrine/paracrine regulatory loop within seminiferous tubules, remain so far unexplored. Our results demonstrate an exclusive expression of Grim-19 in interstitial LCs of rodent testis and unveil a peripheral regulatory hub, involving GRIM-19-mediated phosphorylation of StAR and ligand-binding properties in stimulated LCs of murine testis, as a critical mechanism regulating testicular steroidogenesis.

Studies in testicular tissues, obtained serially along postnatal development, documented a striking profile of expression of testicular Grim-19, with a progressive increase of both mRNAs and proteins from peripubertal period (postnatal day 28, Fig. 1, *B* and *C*), a profile that appears to precede the elevation of testicular NADH-dehydrogenase (NADH-DH) activity that takes place right after 21 days of age, when rodent enters puberty and spermatids start to appear (33). Given that GRIM-19 is present as a core component of NADH-DH/complex I (2) and an impaired NADH-to-NAD+ process catalyzed by mitochondrial NADH-DH is essential for both steroidogenesis (34) and the hypothalamic–pituitary–gonadal axis function (35), our findings are compatible with an eventual stimulatory role of Grim-19 on synthesis of testosterone from (early) pubertal maturation onward. This possibility is in keeping with a recent mass spectrometry study in which Grim-19 has been observed to be involved in the formation of macromolecular protein complexes aiding in the regulation and efficiency of steroid-synthesizing MA-10 mouse LCs (36).

To gain anatomical resolution, immunostaining and immunoblotting studies for mapping the expression of GRIM-19 protein were performed in testicular sections and primary spermatogenic cells, respectively, and depicted an exclusive expression of GRIM-19 in interstitial LCs (Fig. 1). LC-specific expression of testicular GRIM-19 was further verified by immunoblotting analyses after selective elimination of mature LCs by administration of the cytotoxic compound EDS (Fig. S2). Of note, EDS treatment provokes a subsequent wave of proliferation and further differentiation of preexisting undifferentiated LC precursors, which is well known to mimic the normal developmental events of adult-type LCs during puberty (37). Thus, the lack of GRIM-19 expression in testis tissue 7 days following EDS treatment not only confirmed the LC-specific expression of GRIM-19 but also supported the notion that only LCs at advanced stages of differentiation do express GRIM-19. Intriguingly, subcellular localization of GRIM-19 appears not to be unchangeable under different scenarios. GRIM-19 has been shown to localize predominantly in the nucleus of transfected HeLa cells (1) but is present in both cytoplasmic and nuclear compartments of prostate cancer (38) and breast cancer cells (39). In keeping with the previous observations demonstrating mitochondrial expression of GRIM-19 in blastocysts (40) and gastric cancer cells (41), careful analysis of GRIM-19 localization in our study indicated that this protein was mainly present in the mitochondria of LCs (Fig. 1*G*). Altogether, this somewhat contradictory evidence regarding GRIM-19 localization within the cell may actually reflect different roles of this protein in cell biology (42). Compelling data have shown that GRIM-19 is a dual function gene that is involved in mitochondrial metabolism and tumor suppression (42). This functional variability may affect its subcellular expression in a very cell type–specific manner.

In agreement with a unique expression pattern of Grim-19 in LCs during testicular development, a lentiviral vector–mediated inhibition of Grim-19 expression *in vivo* significantly disrupted spermatogenesis by inducing GC apoptosis (Fig. 2, *D* and *E*) and oligozoospermia (Fig. 2, *F*–*H*), and this deleterious effect was largely ascribed to testosterone deficiency since supplement with TP effectively ameliorated reproductive defects in Grim-19 shRNA-treated mice (Fig. 2, *E*–*J*). A tempting explanation for the causal nature of Grim-19 is that it plays a prominent role in mitochondrial oxidative phosphorylation. Indeed, monoallelic loss of Grim-19 causes decline of mitochondrial electron transport machinery viz., respiratory complex (RC)-2, RC-4 and RC-5, and a modest increase in RC-3 levels, thus promoting tumorigenesis in mice (43). Similarly, GRIM-19 point mutation R57H results in RC-1 instability and therefore is associated with the development of hypotonia, dyskinesia, and sensorial deficiencies in human patients (44). As mitochondrial fission and fusion is crucial for steroid hormone synthesis in testicular LCs (45), the available data open up the possibility that Grim-19 may regulate testosterone synthesis directly by maintaining mitochondrial dynamics. Additionally, in contrast to the general belief that mitochondrial GRIM-19 deficiency antagonizes cell apoptosis, GRIM-19 deficiency had no effects on LC viability and apoptosis (Fig. S4). This is totally understandable since GRIM-19 is a core component of mitochondrial complex I, whereas ROS production by complex I in physiological state is relatively humble presenting nonlethal oxidative damage to cells (46).

Mitochondrial respiratory chain is one of the major sources of cellular ROS (46). Therefore, it is a very logical observation that GRIM-19 deficiency triggers oxidative stress in LCs, and GRIM-19 deficiency-influenced phosphorylation landscape of the StAR protein (Fig. 5*I*), as well as reconstruction of ECM-induced activation of Integrin expression, can be effectively abrogated by the pharmacological inhibition of ROS (Fig. 6*A*). Further mechanistic analysis revealed that GRIM-19 deficiency-induced oxidative stress compromised LC function mainly *via* modulating two biochemical events. On one hand, GRIM-19 appears to operate as central posttranslational modification link between stimulated steroidogenesis and StAR expression in LCs, by changing the phosphorylation state of the StAR protein. StAR predominantly mediates the rate-limiting step in steroid biosynthesis, that is, the transport of the substrate of all steroid hormones, cholesterol, from the outer to the inner mitochondrial membrane, thus regulating fundamentally the synthesis of testosterone in LCs (47). The molecular switch from a repressive to a permissive configuration of StAR is seemingly dictated by the hormonal influence on StAR activity at posttranslational level: phosphorylation of StAR not only potentiates its steroidogenic activity (23, 48) but also determines its localization in mitochondria (24) in db-cAMP or hCG-stimulated cells. In our study, the db-cAMP-induced phosphorylation of StAR was totally abolished in the presence of Grim-19 depletion (Fig. 4*G*), and reciprocal coimmunoprecipitation experiments further demonstrated that there was no direct interaction between StAR and GRIM-19 (Fig. 4*H*). These findings indicate that the phosphorylation of StAR is also subjected to GRIM-19-mediated posttranslational regulation during gonadotropin-stimulated steroidogenesis, probably in an indirect manner. On the other hand, its gradually recognized association of LCs with ECM initiates an extensive cell matrix crosstalk, which regulates fundamentally cell adhesion, activation of various second messengers, and testicular steroidogenesis (26, 49, 50). As yet, how the response of LCs to ECM is modulated remains ill-defined. We observed that GRIM-19 deficiency-induced oxidative stress may regulate response to ECM components (namely laminin) by controlling expression of distinct transmembrane α and β subunits in LCs. Ablation of GRIM-19 or treatment with H_2_O_2_ alone promoted α6β1 activation, whereas application of the ROS scavenger NAC totally blocked α6β1 activation in LCs (Fig. 6, *B* and *E*). In favor of our hypothesis, cell adhesion and integrin expression have been shown to be modulated by oxidative stress in EA.hy 926 cells (51). In addition, on MA-10^Grim-19−/−^ cells, both α6 and β1 integrins contribute to adhesion to selected ECM proteins (*i.e*., laminin, Fig. 6*F*). Laminin has been shown to suppress progesterone production by human luteinizing granulosa cells *via* interaction with α6β1integrins (52). Thus, the available data support the general laminin-α6β1 integrins interacting mechanism by which progesterone production is disrupted in GRIM-19-deficient LCs.

**Figure 7:**
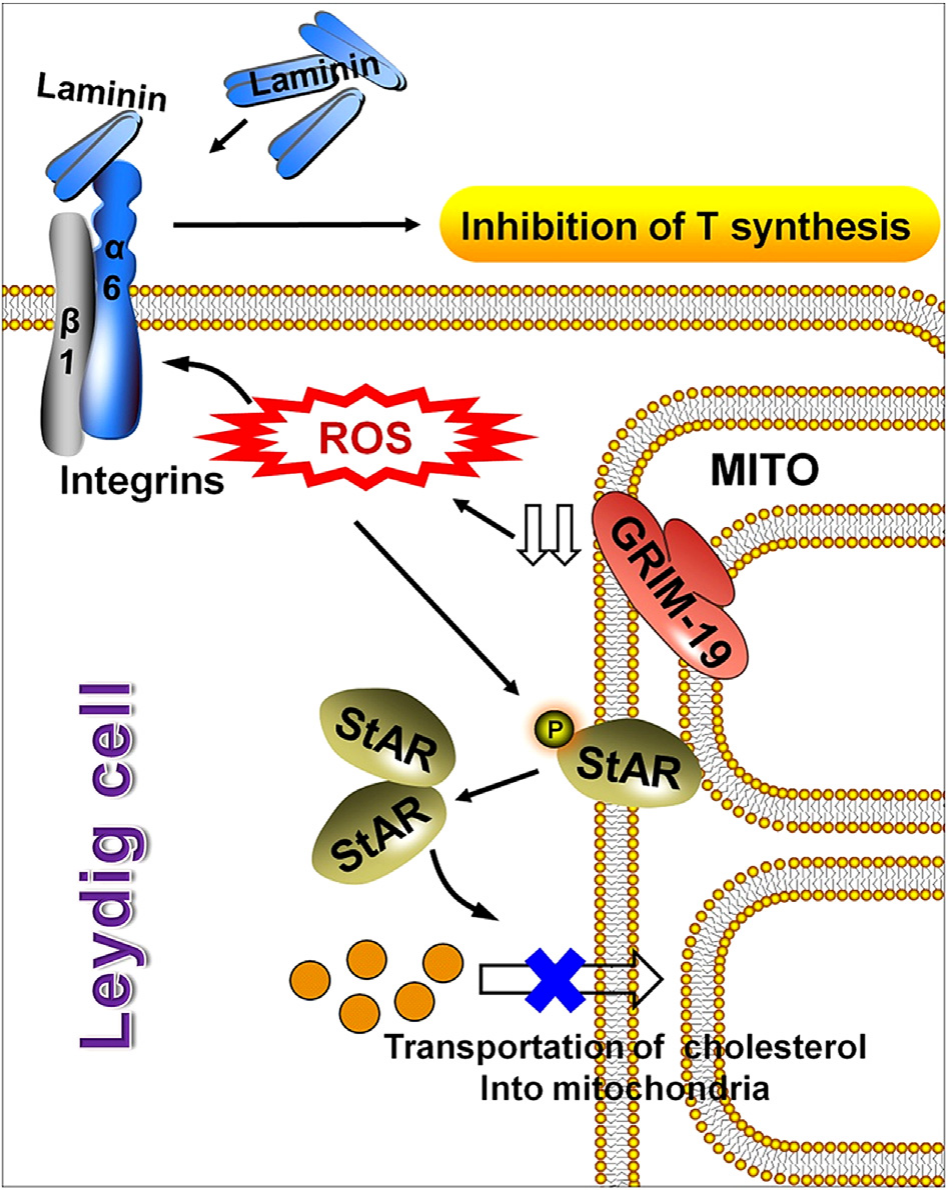
**Summary diagram of the possible mechanisms related to GRIM-19 deficiency contributing to steroidogenic dysfunction.** The negative regulation exerted by GRIM-19 deficiency-induced oxidative stress on steroidogenesis may be the result of two phenomena. A direct effect through inhibition of phosphorylation of StAR and subsequent impediment to StAR localization in mitochondria, and an indirect pathway that is to facilitate the inhibiting role exerted by the ECM on the steroidogenic capacity of LCs *via* promotion of integrin activation. ECM, extracellular matrix; LC, Leydig cell.

Collectively, our data raise the hypothesis that the negative regulation exerted by GRIM-19 deficiency-induced oxidative stress on steroidogenesis is the result of two phenomena. A direct effect through inhibition of phosphorylation of StAR and subsequent impediment to StAR localization in mitochondria, and an indirect pathway that is to facilitate the inhibiting role exerted by the ECM on the steroidogenic capacity of LCs *via* promotion of integrin activation (Fig. 7). Future studies will have to be designed to determine the soundness of such a hypothesis. Moreover, because GRIM-19 also functions as a potent regulator of energy metabolism (43) and because sensitivity to metabolic cues in LCs exert a major influence on spermatogenesis and biosynthesis of testosterone (53), failure of testis GRIM-19 expression to decline in a progressive fashion might contribute to the well-known exacerbation of androgen deficiency in aged males (54), some of which occur in the context of obesity (55) or diabetes (56).

## Experimental procedures

### Animal work

Animal work was complied with the institutional guidelines and the criteria outlined in the “Guide for Care and Use of Laboratory Animals” (NIH publication 86-123), and was approved by Institutional Animal Care and Use Committee (IACUC) of the First Affiliated Hospital of Sun Yat-sen University under protocol #SYSU 2018-041-2b and IACUC of Air Force Medical University (Approval #: KY20194015). Pregnant C57BL/6 mice at 14 days of gestation and adult Sprague–Dawley rats at 4 months of age were purchased from the Animal Research Center of Sun Yat-sen University and were housed under a constant 12 h light:12 h darkness cycle (lights on at 0800 h) and controlled conditions of humidity (between 70% and 80%) and temperature (22 ± 1 °C), with free access to pellet mouse chow and tap water. Mice were allowed to acclimatize at least for 7 days before experiments (57). At 5, 14, 21, 28, 45, 50, and 70 days post-partum, mice were euthanatized by pentobarbital anesthesia (0.04–0.05 mg/g body weight, intraperitoneally ) followed by cervical dislocation. Testes were either fixed in Bouin’s/4% paraformaldehyde solution and embedded in paraffin for histological analysis or snap-frozen in liquid nitrogen and stored at −80 °C for biochemical analysis.

*In vivo* knockdown of Grim-19 was accomplished by using a previously validated lentiviral vector–mediated gene transfer procedure (58, 59). Following pentobarbital anesthesia, testes were exposed under a Leica EZ4 dissecting microscope (Leica) and were injected with 20 μl of pLV-Grim-19 sh-eGFP or scramble shRNA (7 ng of viral capsid proteins/μl, Inovogen) by gentle syringe pressure using a fiber optics probe (diameter, 1.65 mm). The testes were then gently put back into scrotum, muscle and skin layers were sutured accordingly, and animals were allowed to recover (n = 7/group). Two days after lentiviral injection, some mice were injected subcutaneously with 3 mg/kg TP (Sigma–Aldrich) every day for consecutive 68 days. Mice were euthanatized at day 71 after lentiviral injection, and testes and blood samples were collected accordingly.

Detailed information on other procedures pertinent to this article can be found in supporting information.

## Data availability

The authors confirm that the data supporting the findings of this study are available within the article [and/or] its supplementary materials.

## Supporting information

This article contains supporting information (15, 16, 17, 18, 57, 60, 61, 62, 63, 64, 65, 66, 67, 68).

## Conflict of interest

The authors declare that they have no conflicts of interest with the contents of this article.
