# Supplementary material for: Grim-19 plays a key role in mitochondrial steroidogenic acute regulatory protein stability and ligand-binding properties in Leydig cells
Source: J Biol Chem. 2022 Nov 2;298(12):102671. doi: 10.1016/j.jbc.2022.102671 (PMC9768377; doi:10.1016/j.jbc.2022.102671)
Supplement: Supporting information [file mmc1.docx]

**SUPPORTING INFORMATION**

**EXPERIMENTAL PROCEDURES**

***Cell treatment***

The isolation and purification of mouse LCs were performed according to previous reports (1). Seminiferous tubules dispersed from decapsulated testes of adult mice were incubated in a shaking water bath at 34ºC for 20 min with DMEM/F-12 medium containing 0.25 mg/ml Type II collagenase (Sigma-Aldrich). The cell mixture was then filtered through two layers of 100-μm nylon mesh (Thermo Fisher Scientific, Shanghai, China) and centrifuged at 250×g for 5 min. The resultant cell mixture was resuspended in 55% isotonic Percoll (Sigma-Aldrich), followed by density gradient centrifugation at 20,000×g for 60 min at 4 ºC. Leydig cell fractions with densities between 1.070–1.088 g/ml were finally collected, washed with Hanks’ buffered saline, and cultured in DMEM-F12 in a 37 ºC, 5% CO_2_ humid incubator until use. The purity of primary LCs, monitored by histochemical staining of 3beta-hydroxysteroid dehydrogenase (3β-HSD) (2), was >= 95%. MA-10 LCs, obtained from ATCC (Manassas, VA, USA), were cultured in ATCC-formulated DMEM/F-12 Medium containing 15% (v/v) heat-inactivated horse serum and 40 µg/ml of gentamicin. Before conducting experiments, pretreatment of confluent cell layers in culture dishes were washed with PBS and replenished with serum-free DMEM/F-12 medium. All cells used in the current study had less than 20 passages. Primary germ cells (GCs) and SCs were isolated and purified from 8-week-old male C57BL/6J mice, as described by our previous work (3-5).

To study the stimulated steroidogenesis, primary LCs or MA-10 cells were challenged for 12 h with 100 ng/ml luteinizing hormone (LH), 100 ng/ml human chorionic gonadotropin (hCG), 1 mM dibutiryl-cAMP (db-cAMP), 5 μM 22R-hydroxycholesterol (22-ROH), or 5 μM pregnenolone respectively (all reagents were obtained from Sigma-Aldrich) (6), followed by measurement of testosterone or progesterone concentrations in culture media as describe above. To transiently knockdown the expression of *Grim-19*, primary LCs were transfected with *Grim-19* shRNA or Scramble shRNA (Santa Cruz Biotechnology, Shanghai, China) for 48 h, using Lipofectamine® 3000 (Thermo Fisher) according to the manufacturer’s instructions. To establish the MA-10 cell line that was stably deprived of *Grim-19* expression, MA-10 cells were transfected with *Grim-19* shRNA or Scramble shRNA for 48 h using Lipofectamine® 3000. Cells were then split at a ratio of 1:10, followed by incubation in DMEM:F12 supplemented with 2 μg/ml of puromycin (Sigma-Aldrich). After approximate 5~7 days of selection, resistant colonies stably transfected with *Grim-19* shRNA were harvested and designated as MA-10^Grim-19-/-^ cells. The efficiency of shRNA knockdown in LCs was monitored with immunoblotting as described below. To reveal the functional meaning of activation of StAR in GRM-19-regulated steroidogenesis, MA-10^Grim-19-/-^ cells were transfected with a mouse StAR ORF expression plasmid (Sino Biological, Beijing, China) with Lipofectamine® 3000 for 48 h prior to other assays.

***Generation of rodent models***

Adult Sprague-Dawley rats at 4 months of age were purchased from the Animal Research Center of Sun Yat-sen University, and were housed under a constant 12 h light:12 h darkness cycle (lights on at 0800 h) and controlled conditions of humidity (between 70 and 80%) and temperature (22±1°C), with free access to pellet mouse chow and tap water. Selective elimination of mature LCs was achieved using the administration of cytotoxic drug EDS (Sigma-Aldrich, Shanghai, China) in rats (7). Briefly, rats were injected i.p. with a single dose of EDS (in a single dose of 75 mg/kg weight) or vehicle control (dimethyl sulfoxide, water; 1.5:3.5, vol/vol). Rats were euthanatized at 0, 7, 14 and 28 days after EDS administration (n=7/timepoint).

***Antibody pre-absorption***

Where appropriate, pre-absorbed serum was prepared by incubating the anti-GRIM-19 antibody with the GRIM-19 peptide antigen (4A Biotech, final concentration of peptide 1.0 or 5.0 μg/ml) for 12 h at 4°C while rotating, after which the antibody was collected following mild centrifugation (900 ×g).

***Assessment of male fertility, cauda epididymal sperm parameters and testicular apoptosis***

At 70 d after lentiviral injection, mice were subjected to fertility tests as described elsewhere (8). Briefly, one male was housed with two wild-type females. Once copulation plugs were observed the other day, females were immediately removed and were then caged individually, and other females were added. Males were set up with females for a total of 18 days. To evaluate changes in caudal epididymal sperm parameters, The right caudal epididymis was carefully dissected and placed in 0.1 ml of motile buffer (120 mM NaCl, 5 mM KCl, 25 mM NaHCO_3_, 1.2 mM KH_2_PO_4_, 1.2 mM MgSO_4_ and 1.3 mM CaCl_2_). Tissues were gently minced using sterilized surgical scissors and were then placed for 5 min at 37 °C to allow sperm dispersal. The supernatants containing sperm suspension was finally collected and sperm suspension was diluted 25-fold before preparing the counting chamber. Subsequently, a total of 20 grids were counted and sperm density in a 0.1-μl suspension was determined accordingly. Testicular apoptosis was measured with the aid of an apoptosis enzyme-linked immunosorbent assay (ELISA) kit (Roche, Shanghai, China) (9).

***Hormone assay***

Rat/mouse blood samples were harvested between 09:00 and 10:00 a.m. Following anesthesia, rat/mouse blood samples were collected from orbital sinus and transferred to an anticoagulant-free sterile tube. The tubes were placed in a standing position and waited for 30 min, followed by centrifugation at 4ºC at ~ 500 ×g for 5 min. The supernatants containing serum on the top of tube were then collected. Testosterone concentrations in mouse serum as well as testosterone/progesterone concentrations in culture media were determined using a Testosterone ELISA Kit (Abcam, Shanghai, China) and a Progesterone ELISA Kit (Novus, Shanghai, China) respectively, as per the manufacturer’s instructions. The sensitivity of testosterone and progesterone assays is 0.07 ng/ml and 0.2 ng/ml, respectively. The intra-assay precision of these results is <=5.8%, and the inter-assay precision of results is <=10.5%.

***Cell adhesion***

The 96-well culture plates were coated with different concentrations of fibrinogen (2 μg/ml), fibronectin (10 μg/ml), vitronectin (5 μg/ml), plasminogen (10 μg/ml), Cyr61 (2.5 μg/ml), Collagen IV (5 μg/ml), Laminin (5 μg/ml) and VCAM-1 (5 μg/ml) for 3 hours at 37°C. The wells were then post-coated with 0.5% PVA for 1 hour at 22°C (10). For adhesion assays, aliquots (50 μl) of 5×10^5^/ml MA-10 cells in DMEM/F-12 were added to the wells. Cells were challenged for 12 h with 1 mM db-cAMP, followed by incubation for 30 minutes at 37°C. The nonadherent cells were removed by 2 washes with PBS and fluorescence was measured using a CytoFluor II fluorescence plate reader (Applied Biosystems, Shanghai, China).

***Cell viability and apoptosis***

Primary LCs prepared from different testes were seeded in 96-well plates at a density of 5.0×10^4^ cells/well. Following cell culture for 24 or 48 h, the cells were cultured in serum-free medium containing 3-(4,5-dimethyl-thiazol-2-yl)-2,5-diphenyltetrazolium bromide (MTT, Sigma-Aldrich, 0.5 mg/ml), and the plates were incubated at 37°C for another 4 h. Subsequently, 100 μl of DMSO was added to dissolve the formazan crystals and the absorbance was determined in an ELISA reader at 570 nm. The number of metabolically competent cells was determined as the ratio (expressed as a percentage) of absorbance of LCs from Grim-19 shRNA-treated testis to LCs from Scramble shRNA-treated testis that served as a control. Cell apoptosis was measured using an apoptosis ELISA kit (Roche Diagnostics, Shanghai, China), with final spectrophotometry being carried out at 405 nm (11).

***Reactive oxygen species (ROS) measurement***

Intracellular ROS was assayed using 5, and 6-chloromethyl-2,7-dichlorodihydrofluorescein diacetate ethyl ester (DCFH-DA) (12). Primary LCs isolated from different testes were cultured in 48-well plates with 250 μl of phenol red-free DMEM/F12 containing 20 mM HEPES, 50 μg/ml gentamicin. Each well then received 250 μl of the same medium containing 5 μM DCFH-DA (Thermo Fisher Scientific, Shanghai, China). Following a 30-minute culture, cells were challenged for 12 h with 1 mM db-cAMP, followed by measurement of fluorescence under a Nikon TE-300 fluorescent microscope (Nikon, Shanghai, China). Mitochondrial ROS (mROS) was measured using 5.0 μM MitoSOX Red probe (Thermo Fisher Scientific) according to the manufacturer's instructions. Primary LCs were challenged for 12 h with 1 mM db-cAMP, followed by incubation with 5.0 μM MitoSOX at 37°C for another 20 minutes. Final fluorescence was observed under a Nikon TE-300 fluorescent microscope.

***Intracellular reduced glutathione (GSH)/oxidized glutathione (GSSG) ratio and NADP+/NADPH ratio analysis***

The primary LCs isolated from Grim-19 shRNA or Scramble shRNA-treated testes were challenged for 12 h with 1 mM db-cAMP, followed by measurement of intracellular reduced glutathione (GSH)/oxidized glutathione (GSSG) ratio and NADP+/NADPH ratio using commercial kits from Beyotime (Haimen, China), according to the manufacturer’s instructions.

***Quantative real-time RT-PCR (RT-qPCR)***

Total RNA was extracted from testes or LCs using the RNeasy Mini Kit (QIAGEN Inc., Valencia, CA, USA). Following a routine DNase treatment (1 U DNaseI/μg RNA, Applied Biosystems/Ambion, Austin, TX, USA) to remove genomic DNA contamination, ~2 µg of total RNA was subjected to cDNA synthesis using the SuperScript® VILO™ cDNA Synthesis Kit (Thermo Fisher) in a total volume of 20 µl. Reverse transcription reactions were incubated at 65°C for 5 min and at 42°C for 1 h, and were terminated by heating at 70°C for 15 min. Polymerase chain reaction (PCR) analysis was then performed using primers listed in **Supplementary Table 1**. Expression value from the housekeeping gene glyceraldehyde-3-phosphate dehydrogenase (*Gapdh*) gene was used for normalization (9). The PCR amplification profile was: initial denaturation at 94°C for 5 min, 32 cycles of denaturation at 94°C for 30 sec, annealing at 60°C for 40 sec and extension at 72°C for 30 sec and a final extension at 72°C for 10 min in the total volume of 25 μl. PCR-generated DNA fragments were resolved in Tris-borate buffered 1.5% agarose gels and visualized by ethidium bromide staining (1.25 μg/ml, Sigma-Aldrich). To verify changes in gene expression observed by final-time RT-PCR, real-time PCR (qPCR) was performed in selected experimental groups using the fluorescent dye SYBR green I and 1× PCR Master Mix (Applied Biosystems) containing 300 nM of forward and reverse primers, in a final volume of 25 μl, on the MiniOpticon™ Instrument (Bio-Rad Laboratories, Inc., Hercules, CA, USA). Product purity was confirmed by running final products in Tris-borate buffered 1.5% agarose gels and no-template controls were included in all assays, yielding no consistent amplification. Standard curves were constructed for *Grim-19* (specific target) and *Gapdh* (internal control) by plotting values of CT (the cycle at which the fluorescence signal exceeds background) versus log cDNA input (in nanograms). Accordingly, CT values from each experimental sample were then used to calculate the amount of *Grim-19* and *Gapdh* mRNAs relative to the standard. For each sample, results in terms of *Grim-19* expression levels were normalized to those of the internal control *Gapdh*.

***Immunoblotting***

Lysates were prepared in ice-cold RIPA buffer (Tris-HCl 50 mM, NaCl 150 mM, Triton X-100 1% vol/vol, sodium deoxycholate 1% wt/vol, and SDS 0.1% wt/vol pH 7.5) supplemented with complete proteinase-inhibitor cocktail tablets (Roche Diagnostic, Shanghai, China). The protein concentration was then determined using the Pierce™ BCA Protein Assay Kit (Thermo Fisher) according to the manufacturer’s instructions. For immunoblotting analysis, 30 µg proteins was separated on 10-15% SDS/PAGE, followed by transfer to PVDF membrane (Millipore, Bedford, MA, USA) which was subsequently blocked with 5% nonfat milk and 0.1% Tween-20 in Tris-buffered saline, pH 7.4, for 1h at room temperature. Membranes were then incubated with primary antibodies (**Supplementary Table 1**) in blocking solution overnight at 4°C. The membrane was then rinsed with PBS and incubated with horseradish peroxidase conjugated second antibody (**Supplementary Table 1**) for 1 h at room temperature, followed by three rinses with PBS. The horseradish peroxidase was finally visualized by using a chemiluminescence substrate (ECL plus Western blot detection system, Pierce Biosciences, Thermo Fisher). Densitometric scanning of immunoblots was performed using the Image J software (National Institutes of Health) and normalized for the TUBULIN staining (13).

***Morphological examination***

Immunofluorescence staining was performed as described previously (8). Briefly, after deparaffinization and rehydration, 4% paraformaldehyde-fixed sections were incubated with blocking solution (10% donkey serum, 0.5% BSA and 0.3% Triton X-100 in PBS) at room temperature for 1 h. Sections were then incubated with the primary antibodies as indicated (**Supplementary Table 1**), in a humidified chamber overnight at 4°C. Slides were rinsed 3 times with diluent and incubated for approximately 1 h at room temperature with fluorescein isothiocyanate (FITC)/Cy3-labeled second antibodies (**Supplementary Table 1**). Sections were then washed 3 times with diluents, counterstained for 5 minutes with 2-(4-Amidinophenyl)-6-indolecarbamidine dihydrochloride (DAPI, Sigma-Aldrich), and were viewed by epifluorescence with an Axio Imager M1 microscope (Zeiss, Beijing, China). Sections incubated with a preabsorbed serum or nonspecific IgG, instead of the primary antibody (Sigma-Aldrich), were served as negative controls.

For cytochemical immunofluorescence staining, primary LCs were labeled with 500 nM MitoTracker Red CMXRos (Thermo Fisher) in fresh culture media at 37°C for 30 min, and were then fixed with 4% paraformaldehyde for 15 min followed by incubation with blocking solution (10% fetal bovine serum, 0.2% triton X-100 in PBS) and treated with GRIM-19 antibody at dilution of 1:200 at 4°C overnight. The following incubation of second antibody was carried out as described above. Cells were finally observed and recorded under a Zeiss 510 confocal microscope equipped with 347-, 488-, and 543-nm laser beams.

Immunohistochemistry was carried out as described in our previous work (3). Briefly, 5-μm-thick testicular sections were deparaffinized, rehydrated, and subjected to sequential antigen retrieval (20 min at 95°C in 10 nM citrate buffer, pH=6.0) and elimination of endogenous peroxidase activity (20 min at room temperature in 0.5% v/v H_2_O_2_/methanol). Following PBS rinse, slides were incubated at 4°C overnight with different primary antibodies (**Supplementary Table 1**). Subsequent treatment with second antibody and avidin–biotin complex (ABC) was performed with the aid of the VECTASTAIN ELITE® ABC Kit (Vector Lab, Burlingame, CA, USA). Final peroxidase reaction was achieved be incubating sections with 0.7 mg/ml 3-3’-diaminobenzidine tetrahydrochloride (Sigma-Aldrich) in a urea hydrogen peroxide solution (1.6 mg/ml). Slides were counterstained slightly with hematoxylin for 30s to visualize nuclei.

***Cell fractionation and mitochondrial isolation***

The preparation of whole-cell lysates, as well as separation of cytoplasmic and nuclear proteins was performed using Thermo Scientific Subcellular Protein Fractionation Kit exactly according to the manufacturer’s instructions. The isolation of mitochondria was carried out as previously described (14). Briefly, primary LCs were washed with PBS and collected in TSE buffer (0.25 M sucrose, 10 mM Tris [pH 7.4], and 0.1 mM EDTA) containing 1 µg/ml of protease inhibitor cocktail. The cells were then lysed with a Potter Elvehjem homogenizer by 35 passes at 1000×g. The initial lysates were centrifuged twice at 600×g for 30 min at 48°C, with the supernatants transferred to clean tubes after each step. The resultant supernatants were again transferred to clean tubes and centrifuged at 12,000×g for 30 min at 48°C to pellet the mitochondria. The resulting mitochondrial pellets were then resuspended in NET-2 buffer and subjected to following immunoblotting analysis. C-Jun, β-TUBULIN and Cytochrome c were employed as specific markers of nuclear, cytoplasmic and mitochondrial fractions, respectively.

***Northern blotting***

Following db-cAMP treatment, total RNA was prepared and purified from MA-10 cells and cDNA was synthesized with the SuperScript® VILO™ cDNA Synthesis Kit, as described above. The primers used for RT-PCR reaction was 5’-ACAGTGGTGGCCCAGCGAGA-3’ and 5’-CCTGCCTCTGCCTCCCGAGT-3’ (NCBI Reference Sequence: NC_000074.7). The PCR-amplified products were ligated into the pGEM vector (Promega, Beijing, China). Subsequent generation of sense and antisense cRNA probes was achieved by *in vitro* transcription using bacteriophage T7 RNA polymerase provided by a DIG RNA Labeling Kit (Roche). For northern blotting, ~15 μg of RNA samples were electrophoresed on denaturing agarose gel. The separated fragments were then transferred onto a Blotting-Nylon 66 membrane (Sigma-Aldrich). Subsequent hybridization with the DIG-labeled cRNA probes and visualization of positive signals were performed according to the manufacturer’s instructions (BrightStar™ BioDetect™ Nonisotopic Detection system, Thermo Fisher Scientific).

***Immunoprecipitation (IP)***

His-Grim19 and HA-StAR (Sino Biological) were cotransfected into NIH/3T3 cells using Lipofectamine® 3000. 48 h after transfection, NIH/3T3 cells were collected. In another experimental setting, primary LCs isolated from Grim-19 shRNA or Scramble shRNA-treated testes were stimulated with 1 mM db-cAMP for 12 h prior to collection. NIH/3T3 and primary Leydig cells were washed twice with ice-cold PBS, and were lysed with lysis buffer (20 mM Tris, pH 7.4, 150 mM NaCl, 1 mM EDTA, 1 mM EGTA, 1% TritonX-100, 2.5 mM sodium pyrophosphate, 1 mM Na_3_VO_4_, 1 μg/ml aprotinin, 1 μg/ml leupeptin, and 1 mM phenylmethylsulfonyl fluoride) at 4 °C for 30 min, followed by centrifugation at 5,000×g for 10 min to remove cell debris. The resultant cell lysates were then incubated with different primary antibodies (**Supplementary Table 1**) at 4°C overnight. The next day, protein A Sepharose (Pierce, Rockford, IL, USA) was added into lystates and the compound was incubated at 4°C for another 2 h. Immunocomplexes were finally eluted from the sepharose beads by boiling in Laemmli sample buffer and subjected to SDS–PAGE of immunoblotting analysis (5).

***Statistical analysis***

Experiments were repeated at least three times, and one representative result from at least three similar results is presented. The significance of the results was determined by using *Student's t-test* or one way analysis of variance (ANOVA) as appropriate, with the aid of GraphPad Prism 7 (GraphPad Software, San Diego, CA, USA). Statistical differences were considered significant at *P* < 0.05. Data were presented as the mean ± S.E.M.

**
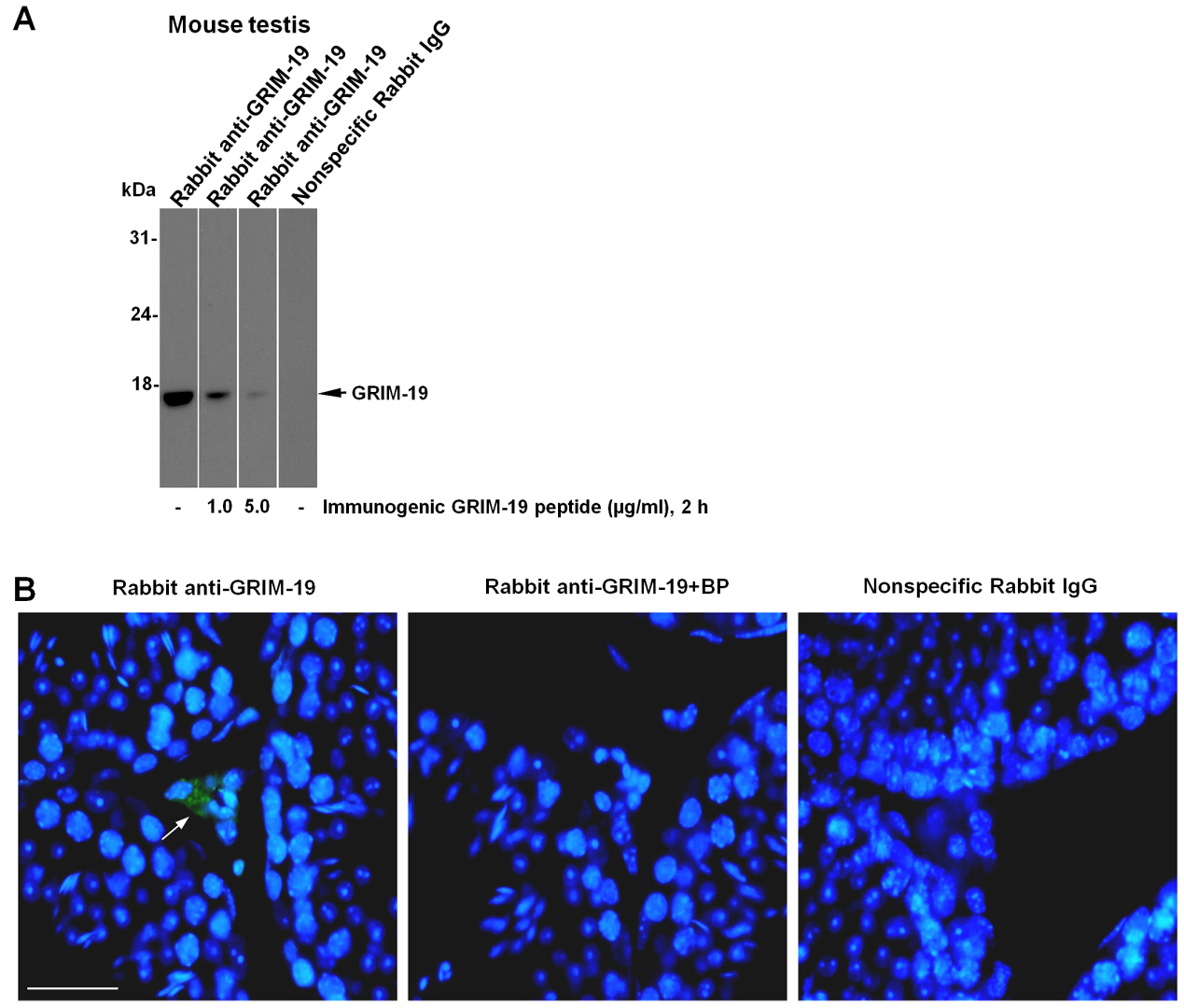
**

**SF. 1** Validation of the specificity of anti-GRIM-19 antibody. (A) Immunoblot stained with an anti-GRIM-19 antibody, a preabsorbed serum prepared by incubation at 4°C for 12 h with different doses of immunogenic GRIM-19 peptides as indicated, or a nonspecific Rabbit IgG, demonstrating the specificity of this antibody. (B) Immunostaining of GRIM-19 (white arrow) in mouse testicular sections were carried out using an anti-GRIM-19 antibody, a preabsorbed serum or a nonspecific Rabbit IgG. Bar=25 μm


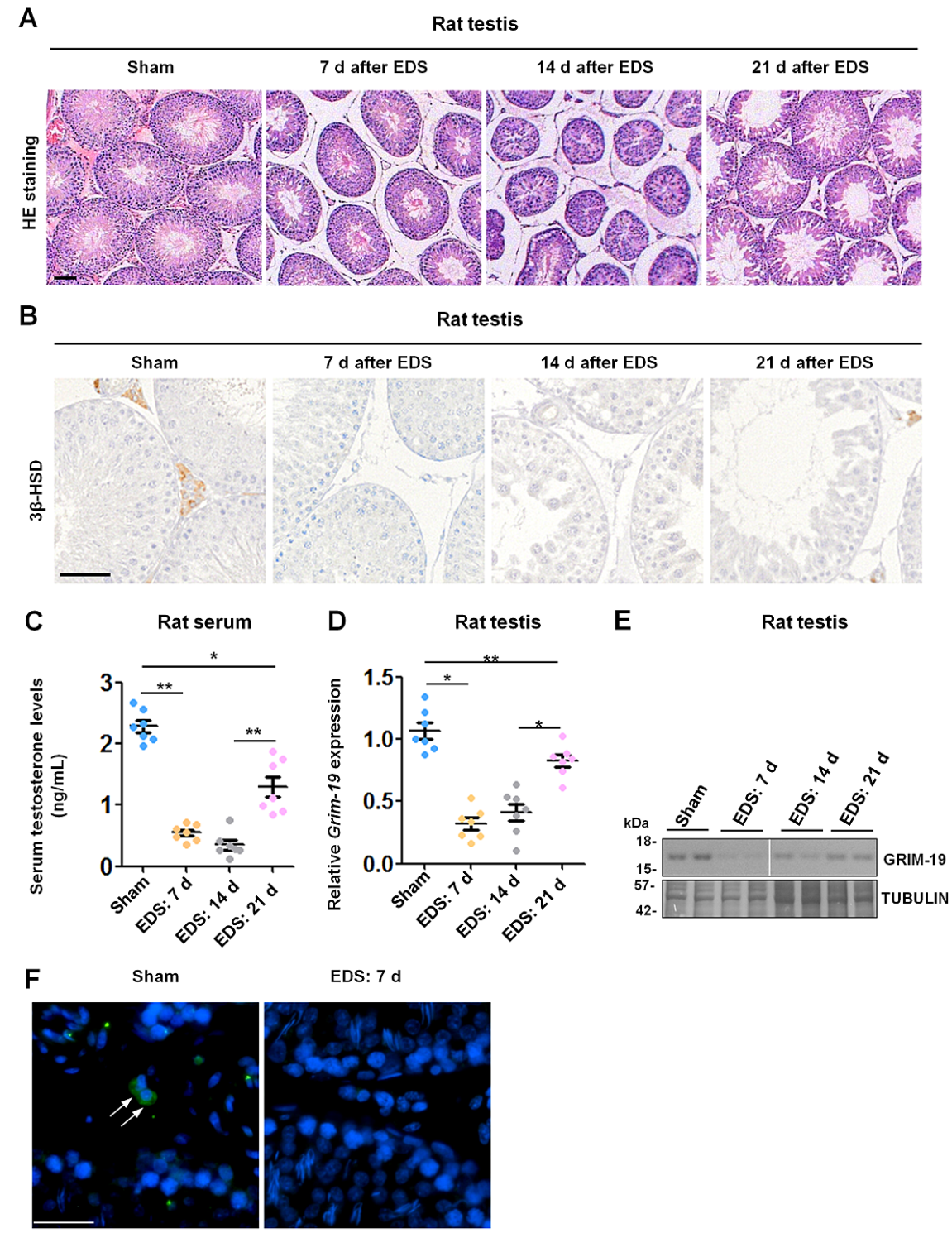


**SF. 2** Validation of the GRIM-19 localization in interstitial Leydig cells (LCs) using a cytotoxic drug ethylene dimethane sulfonate (EDS)-treated rat model. (A) Representative hematoxylin & eosin-stained transverse testis sections showing testicular morphology at 7, 14 and 21 d after administration of the cytotoxic drug EDS. Bar=50 μm (B) 3β-HSD protein expression in rat testis at different time points following EDS treatment was assessed by immunohistochemistry. Bar=50 μm (C) Serum testosterone level (ng/ml) in rats after administration of EDS was evaluated as described in **EXPERIMENTAL PROCEDURES** (**P*<0.05 and ***P*<0.01). (D) Expression levels of *Grim-19* mRNA in rat testis at different time points following EDS treatment were evaluated by RT-qPCR. Data were presented as the mean ± S.E.M. (n=7). (E) GRIM-19 protein expression in rat testis at different time points following EDS treatment was assessed by immunoblotting. TUBULIN was used as the loading control. (F) Immunostaining of GRIM-19 protein in rat testis at 7 d after EDS treatment. White arrows denote GRIM-19 protein in LCs. Bar=25 μm


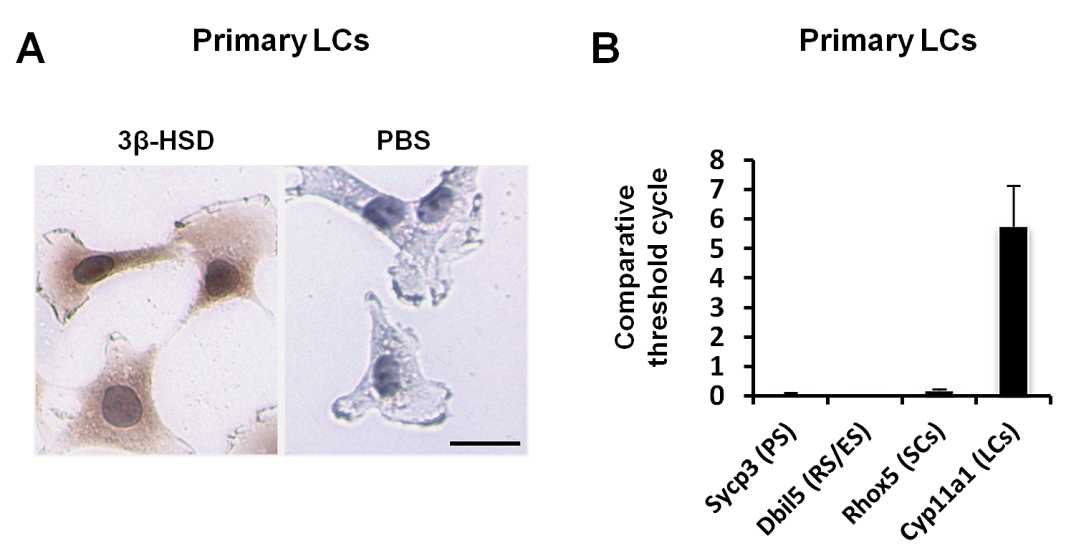


**SF. 3** Verification of the purity of primary LCs isolated from adult mouse testis was carried out using 3β-hydroxysteroid dehydrogenase (HSD3B) immunostaining (A), along with qPCR analysis (B) using primer sets specific to germ cells, Sertoli cells, and LCs marker genes. PS, pachytene spermatocyte; RS/ES, round/elongated spermatid; SCs, Sertoli cells. Bar=10 μm

**
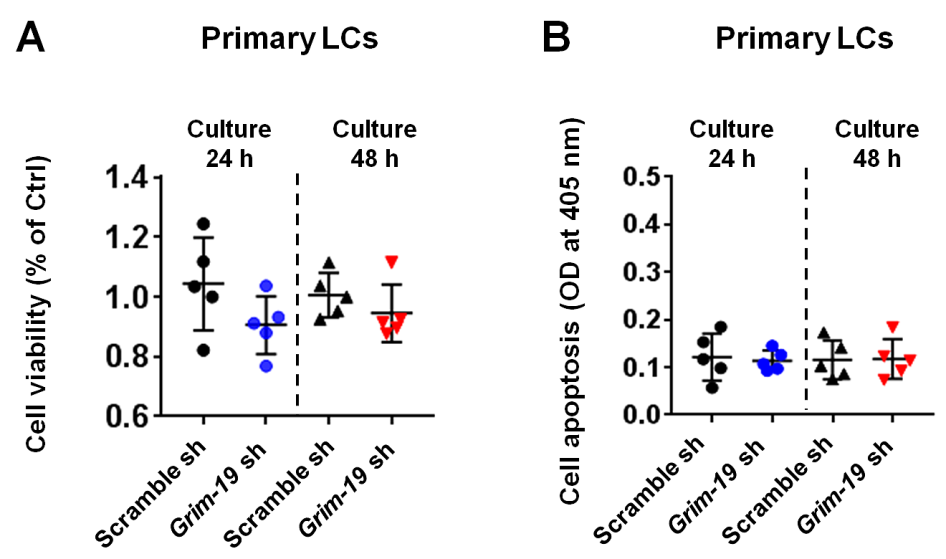
**

**SF. 4** Effects of ablation of GRIM-19 on Leydig cell viability and apoptosis. (A) Primary LCs prepared from different testes were seeded in 96-well plates at a density of 5.0×10^4^ cells/well. Following cell culture for 24 or 48 h, the cells were cultured in serum-free medium containing 0.5 mg/ml MTT, and the plates were incubated at 37°C for another 4 h. Subsequently, 100 μl of DMSO was added to dissolve the formazan crystals and the absorbance was determined in an ELISA reader at 570 nm. The number of metabolically competent cells was determined as the ratio (expressed as a percentage) of absorbance of LCs from Grim-19 shRNA-treated testis to LCs from Scramble shRNA-treated testis that served as a control (n=5). (B) Cell apoptosis was measured using an apoptosis ELISA kit (Roche Diagnostics, Shanghai, China), with final spectrophotometry being carried out at 405 nm (n=5).

| **Supplementary Table 1.** Primers and antibodies used in this study. | | |
| --- | --- | --- |
| **Gene** | **Sequence** | **GenBank#** |
| *Grim-19* Forward | 5’-CTGCCCACTGATGTGGAACA-3’ | NM_023312.3 |
| *Grim-19* Reverse | 5’-TCTTTCGTGAGCCATGGTAGC-3’ |  |
| *Gapdh* Forward | 5’-TACGGGTGCACGTAGCTCA-3’ | NM_008084.3 |
| *Gapdh* Reverse | 5’-AATGAAGGGGTCGTTGATGGC-3’ |  |
| *Ccnd1* Forward | 5’- CCATGGTAGCTGCTGGGAG-3’ | NM_001379248.1 |
| *Ccnd1* Reverse | 5’- CCAGGGCCTTGACCGGG-3’ |  |
| *Loxl4* Forward | 5’-TGCAGAGCAGGAGCTGAAAG-3’ | NM_001164311.1 |
| *Loxl4* Reverse | 5’- GGCTGAGATGAGGTTCCACC-3’ |  |
| *Miox* Forward | 5’- CCTGCGGCCCTACTATCAAG-3’ | NM_019977.2 |
| *Miox* Reverse | 5’- AGCCTAGGTGTACTGGCTCA-3’ |  |
| *Cyp11a1* Forward | 5’- GGGGACAGTATGCTGGCTAAA-3’ | NM_019779.4 |
| *Cyp11a1* Reverse | 5’- AGTAGAGGTACCAGCTCCCTT-3’ |  |
| *Star* Forward | 5’- AGTGGTGTCATCAGAGCTGAAC -3’ | NM_011485.5 |
| *Star* Reverse | 5’- TCAGGTCAATACTGAGCAGCC-3’ |  |
| *Hsd3b1* Forward | 5’- AGTACAGAGGCACAAGCCAG-3’ | NM_008293.4 |
| *Hsd3b1* Reverse | 5’- GGGGCTTAGGGAAGCAAGTT-3’ |  |
| *Pdk4* Forward | 5’- TCACACCTTCACCACATGCT -3’ | NM_013743.2 |
| *Pdk4* Reverse | 5’- AGACGACAGTGGCCTCTACT -3’ |  |
| *Serpina6* Forward | 5’- ATCCCCTGCCAGATGGTACA -3’ | NM_007618.3 |
| *Serpina6* Reverse | 5’- TTAAGTGCAGCGACGACAGT -3’ |  |
| *Nr4a1* Forward | 5’- GGGAGTGTGCTAGAAGGACTG-3’ | NM_010444.2 |
| *Nr4a1* Reverse | 5’- CTTGAATACAGGGCATCTCCAG-3’ |  |
| *Bax* Forward | 5’- CTGGATCCAAGACCAGGGTG-3’ | NM_007527.3 |
| *Bax* Reverse | 5’- GTGAGGACTCCAGCCACAAA-3’ |  |
| *Bcl-xl* Forward | 5’- CTGGTCGCCGGAGATAGATT -3’ | L35049.1 |
| *Bcl-xl* Reverse | 5’- GAGATGGGCTCAACCAGTCC -3’ |  |
| **Antibodies** | **Company** | **Usage** |
| GRIM-19 | Novus Biologicals, NBP1-90050 | IHC-1:100  IF-1:100  IB-1:1000 |
| TUBULIN | Cell Signaling Technology, #2146 | IB-1:3000 |
| C-Jun | Cell Signaling Technology, #9165 | IB-1:2000 |
| Cytochrome c | Cell Signaling Technology, #4272 | IB-1:2000 |
| StAR | Novus Biologicals, NBP1-33485 | IB-1:1000  IP (2μg/reaction tube) |
| HA | ThermoFisher, 14-6756-81 | IB-1:2000 |
| His | ThermoFisher, PA1-983B | IB-1:2000 |
| pho-(Ser/Thr) | Cell Signaling Technology, #9631 | IB-1:500 |
| Goat anti-rabbit FITC secondary antibody | Abcam , ab6717 | IF-1:500 |
| VECTASTAIN® Elite® ABC Kit | Vector Laboratory, PK-6101 | IHC-1:1000 |
| Goat anti-Rabbit IgG -HRP secondary antibody | Abcam , ab97200 | IB-1:1500 |
| Integrin α1 | Santa Cruz Biotechnology, sc-271034 | IB-1:1000 |
| Integrin α2 | Santa Cruz Biotechnology, sc-74466 | IB-1:1000 |
| Integrin α3 | Santa Cruz Biotechnology, sc-374242 | IB-1:1000 |
| Integrin α5 | Santa Cruz Biotechnology, sc-376199 | IB-1:800 |
| Integrin αV | Santa Cruz Biotechnology, sc-376156 | IB-1:1000 |
| Integrin αV/β5 | Santa Cruz Biotechnology, sc-13588 | IB-1:2000 |
| Integrin β1 | Santa Cruz Biotechnology, sc-374429 | IB-1:1000 |
| Integrin β3 | Santa Cruz Biotechnology, sc-365679 | IB-1:1000 |
| Integrin β5 | Santa Cruz Biotechnology, sc-398214 | IB-1:1000 |
| Integrin α6 | Santa Cruz Biotechnology, sc-374057 | IB-1:800 |
| 8-OHdG | Novus Biologicals, NB600-1508 | IF-1:200 |
| Donkey anti-goat Cy3 secondary antibody | Abcam , ab6949 | IF-1:500 |

**References:**

1. Chang, Y. F., Lee-Chang, J. S., Panneerdoss, S., MacLean, J. A., 2nd, and Rao, M. K. (2011) Isolation of Sertoli, Leydig, and spermatogenic cells from the mouse testis. *Biotechniques* **51**, 341-342, 344

2. Zhao, Y., Hou, W. G., Zhu, H. P., Zhao, J., Wang, R. A., Xu, R. J., and Zhang, Y. Q. (2008) Expression of thyrotropin-releasing hormone receptors in rat testis and their role in isolated Leydig cells. *Cell Tissue Res* **334**, 283-294

3. Jin, X., Zhang, S., Ding, T., Zhao, P., Zhang, C., Zhang, Y., and Li, W. (2020) Testicular Lmcd1 regulates phagocytosis by Sertoli cells through modulation of NFAT1/Txlna signaling pathway. *Aging Cell* **19**, e13217

4. Zhang, S., Li, W., Zhu, C., Wang, X., Li, Z., Zhang, J., Zhao, J., Hu, J., Li, T., and Zhang, Y. (2012) Sertoli cell-specific expression of metastasis-associated protein 2 (MTA2) is required for transcriptional regulation of the follicle-stimulating hormone receptor (FSHR) gene during spermatogenesis. *J Biol Chem* **287**, 40471-40483

5. Zhang, C., Lai, J. H., Hu, B., Zhang, S., Zhao, J., and Li, W. (2014) A chromatin modifier regulates Sertoli cell response to mono-(2-ethylhexyl) phthalate (MEHP) via tissue inhibitor of metalloproteinase 2 (TIMP2) signaling. *Biochim Biophys Acta* **1839**, 1170-1182

6. Bi, X., Liu, J., Xu, S., Wang, Y., and Wu, X. (2021) Testicular STAC3 regulates Leydig cell steroidogenesis through potentiating mitochondrial membrane potential and StAR processing. *Cell Tissue Res* **384**, 195-209

7. He, K., Qu, H., Wang, H., Zhang, S., Qian, X. H., and Li, W. (2016) Regulated and Functional Expression of the Corepressor MTA3 in Rodent Testis. *Endocrinology* **157**, 4400-4410

8. Dong, Y. S., Hou, W. G., Li, Y., Liu, D. B., Hao, G. Z., Zhang, H. F., Li, J. C., Zhao, J., Zhang, S., Liang, G. B., and Li, W. (2016) Unexpected requirement for a binding partner of the syntaxin family in phagocytosis by murine testicular Sertoli cells. *Cell Death Differ* **23**, 787-800

9. Li, W., Wu, Z. Q., Zhang, S., Cao, R., Zhao, J., Sun, Z. J., and Zou, W. (2016) Augmented expression of gamma-glutamyl transferase 5 (GGT5) impairs testicular steroidogenesis by deregulating local oxidative stress. *Cell Tissue Res* **366**, 467-481

10. Yakubenko, V. P., Yadav, S. P., and Ugarova, T. P. (2006) Integrin alphaDbeta2, an adhesion receptor up-regulated on macrophage foam cells, exhibits multiligand-binding properties. *Blood* **107**, 1643-1650

11. Liang, Y., Dong, Y., Zhao, J., and Li, W. (2013) YES1 activation elicited by heat stress is anti-apoptotic in mouse pachytene spermatocytes. *Biol Reprod* **89**, 131

12. Tai, P., and Ascoli, M. (2011) Reactive oxygen species (ROS) play a critical role in the cAMP-induced activation of Ras and the phosphorylation of ERK1/2 in Leydig cells. *Mol Endocrinol* **25**, 885-893

13. Liu, F., Chen, Z. Z., Zhao, J., Zhang, Y. Q., Ma, J., and Li, W. (2022) T2DM-elicited oxidative stress represses MTA3 expression in mouse Leydig cells. *Reproduction* **163**, 267-280

14. Ogishima, T., Kinoshita, J. Y., Mitani, F., Suematsu, M., and Ito, A. (2003) Identification of outer mitochondrial membrane cytochrome b5 as a modulator for androgen synthesis in Leydig cells. *J Biol Chem* **278**, 21204-21211
